# Ferulic Acid: Mechanistic Insights and Multifaceted Applications in Metabolic Syndrome, Food Preservation, and Cosmetics

**DOI:** 10.3390/molecules30183716

**Published:** 2025-09-12

**Authors:** Daniel A. Jacobo-Velázquez

**Affiliations:** Tecnologico de Monterrey, Escuela de Ingeniería y Ciencias, Campus Monterrey, Av. Eugenio Garza Sada 2501 Sur, Monterrey 64849, NL, Mexico; djacobov@tec.mx

**Keywords:** ferulic acid, nutraceutical, antioxidant, metabolic syndrome, food additive, cosmeceuticals, prebiotic, lipid oxidation, polyphenols, natural preservative

## Abstract

Ferulic acid (FA) is a plant-derived phenolic compound recognized for its potent antioxidant and anti-inflammatory properties, with growing applications in health, food, and cosmetic sciences. This review presents a comprehensive overview of recent in vivo and clinical evidence, emphasizing its effects on metabolic syndrome (MetS) and its technological applications in food and cosmetics. FA has shown preventive and therapeutic potential in managing MetS by improving glucose and lipid metabolism, lowering blood pressure, reducing oxidative stress, and modulating inflammatory and microbiota-related pathways. In food systems, FA serves as a multifunctional additive, exhibiting antimicrobial activity, inhibition of lipid oxidation, stabilization of pigments, and protection of sensitive nutrients that extend shelf life and enhance nutritional quality. Its inclusion in cosmetic formulations further demonstrates synergistic photoprotective effects with conventional UV filters, antioxidant support for vitamins C and E, and anti-aging and skin-brightening properties, although formulation challenges remain. Collectively, FA’s pleiotropic actions and compatibility with clean-label trends highlight its value as a natural bioactive. Continued research on improving its stability, bioavailability, and clinical validation will support its broader integration into functional food, nutraceutical, and cosmeceutical products.

## 1. Introduction

**Figure 1:**
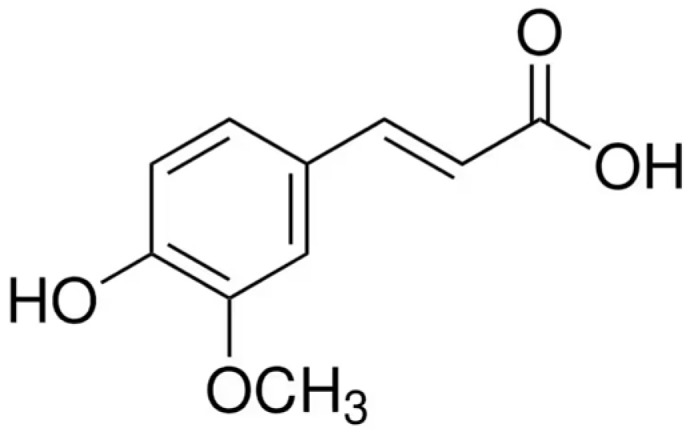
Chemical structure of ferulic acid (4-hydroxy-3-methoxycinnamic acid), a phenolic compound of the hydroxycinnamic acid family.

Ferulic acid (FA; 4-hydroxy-3-methoxycinnamic acid) is a widespread plant phenolic found in cell walls, where it is typically esterified to polysaccharides such as arabinoxylans. Structurally, FA consists of a phenolic ring with methoxy and hydroxy substituents and an α,β-unsaturated carboxylic acid side chain (Figure 1). This configuration stabilizes phenoxyl radicals via resonance, endowing FA with potent antioxidant activity [1]. From a physicochemical perspective, FA is a weak acid (pKa ≈ 4.5) with moderate water solubility, higher in alkaline conditions. It occurs in both free and bound forms and can exist as *trans*- and *cis*-isomers, with the *trans* form being predominant and more stable. These properties influence its extraction, stability, and bioavailability in foods and formulations. In plants, FA contributes to cell wall rigidity by crosslinking lignin and hemicellulose, enhancing resistance to UV radiation and microbial attack [2]. Dietary sources of FA are abundant and include whole grains (notably rice bran, corn, and wheat), certain fruits (e.g., tomatoes, oranges), vegetables (e.g., carrots, sweet corn), seeds, nuts, and beverages such as coffee and beer. In foods, FA is predominantly found esterified to arabinoxylans and other cell wall polysaccharides, with the highest concentrations reported in cereal brans such as rice, wheat, and corn. Fruits and vegetables generally contain lower levels, mostly in free or soluble conjugated forms, whereas beverages like coffee and beer are notable sources of soluble FA. This distribution pattern explains why whole grains and bran fractions are considered the richest dietary reservoirs of FA.

FA occurs in both free and bound forms in foods, the latter released during digestion or fermentation [3,4]. From a technological standpoint, FA is commonly obtained from cereal brans and other plant by-products through alkaline or enzymatic hydrolysis, which cleaves ester linkages to cell wall polysaccharides and releases bound ferulates for further purification. Over the past decade, interest in ferulic acid has grown substantially due to its pleiotropic bioactivities. Numerous in vitro and in vivo studies demonstrate antioxidant, anti-inflammatory, antidiabetic, cardioprotective, and neuroprotective effects of FA [5,6,7,8,9]. These biological activities position FA as a promising nutraceutical for managing chronic diseases, particularly metabolic syndrome (MetS), a cluster of conditions (obesity, insulin resistance, hypertension, dyslipidemia) that elevate the risk of type 2 diabetes and cardiovascular disease. Given the global prevalence of MetS (affecting over one-quarter of adults), multi-targeted dietary strategies are being explored as complementary approaches to traditional pharmacotherapy. Beyond its therapeutic potential, FA is employed in diverse commercial applications.

In the nutraceutical and functional food sectors, it is used both as an active ingredient and as a precursor of γ-oryzanol (a mixture of FA esters with phytosterols) derived from rice bran oil. FA is also present in several traditional herbal remedies and has a strong safety profile, with toxicological studies supporting its use in dietary supplements [10]. In the food industry, FA is gaining attention as a natural preservative due to its ability to inhibit lipid peroxidation and microbial growth, thereby extending shelf life and meeting consumer demand for clean-label additives. It has also shown efficacy as a stabilizer for colorants and vitamins in fortified foods [11].

In the cosmetic sector, FA is widely incorporated into topical antioxidant serums (often alongside vitamins C and E) because of its photoprotective and anti-aging effects. These formulations help neutralize oxidative stress in skin, improving skin tone and reducing UV-induced damage. However, practical challenges such as FA’s poor water solubility and susceptibility to degradation have spurred research into novel delivery systems (e.g., encapsulation in biopolymers, liposomes, nanoemulsions) to enhance its stability and bioavailability in both food and cosmetic applications [12].

This review offers a comprehensive analysis of ferulic acid’s chemistry, bioactivity, and applications. Section 2 discusses FA’s nutraceutical effects against components of MetS, drawing on recent animal models and human studies. Section 3 delves into the mechanistic basis of these effects, including antioxidant, anti-inflammatory, metabolic, vascular, and microbiota-modulating actions. Section 4 examines FA’s role as a food additive, highlighting its preservative function and use in improving nutritional quality and gut health. Section 5 explores FA’s cosmeceutical applications, detailing its role in skin protection and innovative formulation strategies. Finally, we conclude with perspectives on future research directions, including the need for clinical validation, exploration of synergistic combinations, clarification of FA’s metabolic fate in the body, and advancements in biotechnological production and integration of FA into the bioeconomy.

## 2. Ferulic Acid as a Nutraceutical for Metabolic Syndrome

Metabolic syndrome (MetS) is a cluster of interconnected conditions, including central obesity, dyslipidemia, hyperglycemia (insulin resistance/type 2 diabetes), and hypertension, that together elevate the risk of cardiovascular disease and type 2 diabetes. Approximately one-quarter of the world’s adults are estimated to have MetS. There is growing interest in bioactive dietary compounds like ferulic acid as adjuncts for the prevention and management of MetS, due to their multi-targeted actions and relative safety. In this section, evidence from in vivo studies (Table 1) and human trials (Table 2) on the effects of FA against key MetS components is reviewed, along with proposed mechanisms.

### 2.1. Anti-Obesity Effects

#### 2.1.1. Evidence from Animal Studies

Multiple in vitro and in vivo studies indicate that ferulic acid (FA) exerts significant anti-obesity effects in the context of metabolic syndrome (Table 1). In high-fat diet (HFD) animal models, FA supplementation consistently reduces body weight gain and adiposity. For instance, a mouse study showed that FA (0.5 g/kg diet) significantly suppressed HFD-induced increases in body weight, visceral fat mass, adipocyte size, and hepatic steatosis, with efficacy comparable to sibutramine [13]. Alongside these reductions in adiposity, FA-treated mice showed improved serum lipid profiles and glucose control [13].

FA also attenuates obesity-related inflammation: HFD-fed rodents receiving FA exhibited lower levels of proinflammatory cytokines such as TNF-α, IL-6, and MCP-1 in adipose tissue and serum [14]. In one study, dietary FA at 0.5% prevented increases in hepatic lipids and adipocyte hypertrophy, effects associated with reductions in IL-6 and TNF-α [14].

Furthermore, FA favorably modulates metabolic hormone profiles. In obese mice, FA supplementation reversed hyperleptinemia and normalized reduced ghrelin levels; fasting hyperinsulinemia was also decreased, indicating improved insulin sensitivity [13].

FA exerts short-term anorexigenic effects: Halter et al. [27] reported up to 70% acute food intake suppression shortly after FA administration, an effect accompanied by reduced digestive enzyme activity.

Mechanistically, FA activates energy expenditure pathways. FA-treated mice showed increased AMPK phosphorylation and enhanced expression of lipid oxidation enzymes (e.g., CPT1, hormone-sensitive lipase) along with downregulation of adipogenic transcription factors (PPARγ, C/EBPα, SREBP-1c) via MEK/ERK and p38 MAPK modulation [13,23].

Important for obesity-associated gut dysfunction, FA has been shown to modulate intestinal microbiota and barrier integrity. In HFD-fed mice, FA increased short-chain fatty acid (SCFA)-producing bacteria (e.g., *Faecalibaculum*) and reduced endotoxin-producing taxa. These microbiome shifts correlated with increased SCFA receptor expression, reduced plasma lipopolysaccharide (LPS), and downregulated colonic TLR4/NF-κB signaling, suggesting reinforcement of gut barrier function against metabolic endotoxemia [28].

Synergistic strategies combining FA and lifestyle interventions also show promise. In a 2022 13-week study, FA combined with regular exercise in HFD-fed mice led to superior reduction in weight gain, improved lipid profiles, enhanced hepatic antioxidant enzyme activity, and improved exercise performance compared to either treatment alone [22].

#### 2.1.2. Evidence from Human Studies

Human data are still limited. In a six-week randomized, double-blind, placebo-controlled trial, hyperlipidemic adults supplemented with 1000 mg/day FA showed no significant weight loss but exhibited improved lipid profiles, total cholesterol decreased by ~8%, LDL cholesterol by ~9%, and triglycerides by ~12%, and HDL cholesterol increased by ~4%, along with reductions in malondialdehyde (~24%), hs-CRP (~32%), and TNF-α (~13%), without adverse effects [24].

A randomized dietary swap trial in overweight adults replacing white rice with brown rice (high in FA) for 12 weeks produced modest reductions in body weight, BMI, and waist circumference, reflecting observational associations between whole grain intake and obesity risk [23]. Another crossover trial using brewer’s spent grain extract rich in FA found stable weight and improved inflammatory markers in prediabetic participants [23].

### 2.2. Anti-Dyslipidemic and Cardioprotective Effects

#### 2.2.1. Evidence from Animal Studies

Recent studies provide strong evidence that ferulic acid (FA) exerts significant anti-dyslipidemic and cardioprotective effects in both animal models and humans. In high-fat-diet-fed or genetically predisposed rodents, FA supplementation improves serum lipid profiles and mitigates atherosclerotic damage. For example, Gu et al. [15] demonstrated that FA treatment in ApoE^−/−^ mice on a high-fat diet led to lower total cholesterol (TC), triglycerides (TG), and low-density lipoprotein cholesterol (LDL-C) levels, along with a reduced atherogenic index, compared to untreated controls. Importantly, FA-treated mice developed significantly smaller aortic atherosclerotic plaques (with increased collagen content, indicating more stable plaques) similar to the improvements seen with simvastatin therapy. FA also alleviated hepatic steatosis in these mice, as evidenced by reduced liver lipid accumulation, lower serum alanine/aspartate aminotransferases, and decreased oxidative stress markers (malondialdehyde, MDA), while boosting antioxidant defenses (e.g., superoxide dismutase activity). On a molecular level, Gu et al. reported that FA upregulated AMP-activated protein kinase (AMPK) in the liver and downregulated lipogenic enzymes such as sterol regulatory element-binding protein-1 (SREBP-1) and acetyl-CoA carboxylase 1 (ACC1). Through this AMPK/SREBP-1/ACC1 pathway, FA inhibited de novo fatty acid and cholesterol synthesis, explaining its lipid-lowering action in vivo.

Consistent results were observed by Luo et al. [16], who found that FA supplementation attenuated diet-induced hypercholesterolemia in mice by ~13%. This cholesterol reduction was attributed to an increase in hepatic bile acid synthesis via upregulation of cholesterol 7α-hydroxylase (CYP7A1), the rate-limiting enzyme in bile acid production. Notably, FA activated CYP7A1 through an FXR-independent mechanism, leading to greater conversion of cholesterol to bile acids and enhanced fecal bile acid excretion. Such findings reveal a novel pathway by which FA promotes cholesterol catabolism and removal. Other mechanistic studies have identified additional molecular targets: Gao et al. [29] showed that FA binds to and activates long-chain acyl-CoA synthetase 1 (ACSL1), which in turn triggers mitochondrial recruitment of ACSL1 and elevates the AMP/ATP ratio, activating AMPK. The activated AMPK then suppresses the synthesis of triglycerides and cholesterol in diabetic db/db mice, thereby ameliorating lipid metabolic disorders. These multi-pathway effects (enhancing cholesterol elimination and inhibiting lipid synthesis) underscore FA’s therapeutic potential against dyslipidemia [30].

FA’s cardioprotective benefits extend to anti-atherosclerotic and anti-inflammatory actions. A recent study by Hong et al. [17] revealed an innovative mechanism whereby FA combats atherosclerosis by boosting adipose tissue thermogenesis. In ApoE^−/−^ mice, FA (and the related phenolic protocatechuic acid) upregulated uncoupling protein 1 (UCP1) in brown adipose tissue and perivascular adipose tissue, thereby increasing energy expenditure and the “browning” of fat. Treated mice exhibited significantly reduced aortic plaque areas alongside lower expression of pro-inflammatory cytokines (interleukin-1β, interleukin-6, tumor necrosis factor-α) in aortic lesions. The authors further showed that conditioned media from FA-activated brown fat cells could suppress NLRP3 inflammasome signaling in macrophage foam cells, reducing IL-1β secretion and foam cell formation. This suggests that FA can inhibit atherogenesis at least in part by modulating crosstalk between adipose tissue and arterial wall inflammation, ultimately stabilizing plaques. In addition to these in vivo findings, cellular studies reinforce FA’s protective role against oxidative and inflammatory injury in the cardiovascular system. For instance, Wu et al. [31] demonstrated that FA protects macrophages from oxidized LDL-induced cell death by inhibiting the HIF-1α pathway: FA-treated foam cells showed decreased reactive oxygen species, reduced ferroptosis/apoptosis, and increased expression of antioxidant proteins like GPX4. Suppression of HIF-1α prevented downstream inflammatory and cell-death signaling, an effect reversible by HIF-1 agonists. This mechanism aligns with FA’s broader antioxidative and anti-inflammatory profile observed in animal models of atherosclerosis.

#### 2.2.2. Evidence from Human Studies

Importantly, the beneficial effects of ferulic acid observed in preclinical models have also translated to clinical improvements in humans. A landmark randomized controlled trial by Bumrungpert et al. [24] investigated pure ferulic acid supplementation in hyperlipidemic patients. In this six-week trial, subjects receiving 1000 mg/day of FA showed significant reductions in total cholesterol (~8% decrease), LDL-C (~9% decrease), and triglycerides (~12% decrease) compared to placebo, alongside a modest increase in HDL-C. Furthermore, FA supplementation led to a 7.1% decrease in oxidized LDL levels (*p* = 0.002), indicating lowered LDL oxidation, which is a key factor in atherogenesis. Measures of oxidative stress and inflammation were markedly improved: serum MDA declined by ~24% and high-sensitivity C-reactive protein (hs-CRP) by ~32%, while TNF-α was reduced by 13% versus baseline. These changes were all statistically significant and suggest that FA not only improves lipid profiles but also attenuates lipid peroxidation and systemic inflammation in patients. Notably, interleukin-6 (IL-6) was undetectable in this study (likely due to mild inflammation in the sample), but the significant drops in CRP and TNF-α point to an overall anti-inflammatory effect of FA supplementation. By the end of the trial, the FA group had a better cardiovascular risk profile, supporting the idea that FA can be a useful adjunct nutraceutical for managing hyperlipidemia and preventing cardiovascular disease.

Beyond trials of pure ferulic acid, clinical studies of ferulic-acid-rich compounds have reported concordant benefits. Nikooyeh et al. [25], for example, conducted a 12-week RCT in adults with type 2 diabetes using γ-oryzanol-fortified canola oil (γ-oryzanol is a mixture of ferulate esters abundant in rice bran). The group consuming γ-oryzanol-enriched oil showed significant reductions in fasting triglyceride levels (−17.9 mg/dL on average) compared to groups using regular canola or sunflower oil. Moreover, only the γ-oryzanol group experienced notable improvements in cardiometabolic markers such as waist circumference, blood pressure, fasting glucose, and HbA1c. While total cholesterol and LDL-C did not change significantly in this short intervention, the selective drop in TG (along with better glycemic control and blood pressure) highlights the potential of ferulic acid derivatives to favorably influence components of metabolic syndrome in humans. These clinical findings complement the animal and mechanistic data, collectively indicating that ferulic acid can improve lipid metabolism, reduce oxidative LDL damage, and quell inflammation, all of which are crucial for cardiovascular protection.

### 2.3. Anti-Diabetic and Glycemic Control Effects

#### 2.3.1. Evidence from Animal Studies

Extensive in vivo studies indicate that ferulic acid exerts significant anti-diabetic effects in rodent models of diabetes. In streptozotocin (STZ)- or diet-induced diabetic rats, chronic FA treatment leads to lowered fasting blood glucose levels and improved glucose tolerance and insulin sensitivity [18,19]. For example, Salau et al. [18] showed that FA administration (150–300 mg/kg for five weeks in fructose-STZ type 2 diabetic rats) significantly reduced hyperglycemia while elevating serum insulin levels, consistent with an improvement in insulin action. Mechanistically, FA appears to modulate hepatic glucose metabolism in favor of glycemic control. Treated diabetic animals exhibit enhanced hepatic glycogen storage and suppression of gluconeogenesis: FA increases hepatic glucokinase activity and glycogen synthase function (promoting glycogen synthesis), while markedly downregulating the key gluconeogenic enzymes phosphoenolpyruvate carboxykinase (PEPCK) and glucose-6-phosphatase (G6Pase) in the liver [18,19]. In high-fat-diet-induced diabetic mice, FA supplementation prevented the pathological rise in hepatic PEPCK and G6Pase, thereby reducing excess hepatic glucose output and resulting in improved overall glucose homeostasis [19]. This effect has been linked to FA’s ability to activate insulin-dependent signaling in the liver: notably, FA induces phosphatidylinositol-3-kinase/Akt signaling, which in turn phosphorylates and inactivates the transcription factor FoxO1, a key promoter of gluconeogenic gene expression [19]. By inhibiting FoxO1 activity, FA treatment blunts the transcription of PEPCK and G6Pase, contributing to lower hepatic glucose production and improved glycemic control [19]. Consistent with these findings, FA-treated diabetic rodents also show reduced activity of fructose-1,6-bisphosphatase and glycogen phosphorylase, further indicating suppression of gluconeogenesis and glycogen breakdown [18]. Taken together, these results demonstrate that FA can restore a more normal balance of hepatic glucose utilization vs. production in diabetes, thereby lowering blood glucose.

Ferulic acid’s anti-diabetic actions are not limited to the liver. Peripheral insulin-sensitive tissues also respond to FA, partly through enhanced glucose uptake. Studies have reported that FA can activate the PI3K/Akt pathway in skeletal muscle and adipose tissue, promoting the translocation of GLUT4 glucose transporters to the cell membrane and thus facilitating insulin-mediated glucose uptake [20]. In STZ-diabetic rats, chronic FA administration improved insulin responsiveness in the myocardium; Chowdhury et al. [20] observed that FA upregulated GLUT4 levels in cardiac muscle and enhanced GLUT4 translocation to the membrane via PI3K/Akt activation, which helped increase glucose utilization in diabetic hearts [20]. Similarly, in cellular models, FA has been shown to increase GLUT4 expression in muscle cells and adipocytes through a PI3K-dependent mechanism, further supporting its role in improving peripheral insulin sensitivity and glucose uptake [20].

Another important aspect of FA’s anti-diabetic effect is its antioxidant and anti-inflammatory activity in diabetic tissues. Hyperglycemia is known to induce oxidative stress and inflammation, which contribute to insulin resistance and β-cell dysfunction. Ferulic acid, a potent phenolic antioxidant, counteracts these pathogenic factors in diabetes. In vivo, FA supplementation has been found to elevate endogenous antioxidant defenses (e.g., increasing hepatic glutathione levels and activities of superoxide dismutase and catalase) while reducing markers of oxidative damage such as malondialdehyde (MDA) in diabetic animals [18]. By scavenging reactive oxygen species and upregulating antioxidant enzymes, FA protects insulin-producing β-cells and other organs from oxidative injury. Ferulic acid also suppresses pro-inflammatory pathways in diabetes. It has been shown to inhibit the overactivation of NF-κB and to lower pro-inflammatory cytokine levels (e.g., TNF-α, IL-6) in diabetic rodents, thereby alleviating inflammation-linked insulin resistance [18,20]. Notably, ferulic acid’s protective effects on pancreatic islets have been documented in models of both type 1 and type 2 diabetes. FA treatment preserves islet architecture and β-cell viability, associated with reduced oxidative stress in the pancreas and lower incidence of β-cell apoptosis [18]. In a rat model of gestational diabetes, FA (20 mg/kg for 12 weeks) improved maternal glucose control and protected fetal/placental tissues; this was accompanied by upregulation of insulin signaling molecules (phosphorylated IRS-1/IRS-2 and PI3K) and increased GLUT1/GLUT4 expression in insulin-sensitive tissues [23]. Collectively, these animal studies demonstrate that ferulic acid not only lowers blood glucose and improves insulin sensitivity but also addresses the underlying metabolic derangements by modulating key enzymatic pathways (gluconeogenesis, glycogenesis) and mitigating oxidative-inflammatory damage in diabetes.

#### 2.3.2. Evidence from Human Studies

Translating these promising findings to humans, early clinical evidence suggests that ferulic acid can confer metabolic benefits, though robust glycemic improvements in diabetic patients have yet to be firmly established. To date, there have been only a few clinical studies examining isolated ferulic acid in humans, and most are short-term trials in related metabolic conditions. In a randomized, double-blind trial involving hyperlipidemic adults (who mostly had normal baseline glycemia), oral supplementation with ferulic acid at 1 g/day for six weeks significantly improved cardiovascular risk parameters, lowered total and LDL cholesterol by ~8–9%, reduced triglycerides, and increased HDL-C, while also markedly decreasing oxidative stress (MDA levels) and systemic inflammation (with a 33% drop in high-sensitivity C-reactive protein and ~13% reduction in TNF-α) [24]. Notably, in this trial, ferulic acid was well tolerated and produced these antioxidant and anti-inflammatory benefits without any adverse effects. However, fasting blood glucose was not significantly changed by the six-week FA supplementation in these non-diabetic, hyperlipidemic subjects [24]. This suggests that while FA can favorably modulate risk factors (lipids, oxidative stress, inflammation) linked to metabolic syndrome, its direct impact on glycemic control in humans may require longer exposure or diabetic populations to become evident.

Limited data are available on ferulic acid interventions in patients with diabetes. One dietary intervention study tested FA-rich whole grains in overweight individuals at risk of metabolic syndrome: Costabile et al. [26] provided a wheat-aleurone-rich diet (naturally enriched in ferulic acid and other phytochemicals) to participants for eight weeks. Consistent with FA’s antioxidant role, the aleurone diet group showed improved oxidative stress markers compared to control; however, there were no significant improvements in fasting or postprandial glucose, insulin levels, or other glycemic measures in these overweight subjects [26]. The authors noted that, despite the strong anti-oxidative effects observed, the addition of ferulic-acid-rich cereals was insufficient to alter glucose homeostasis or insulin sensitivity over the study period [26]. These human findings highlight a discrepancy with the animal studies and underscore the need for more clinical research. It is possible that ferulic acid’s glycemic effects in humans are subtle or require specific conditions (such as overt diabetes or longer duration) to manifest. Importantly, the existing trials did not report any adverse glycemic effects, indicating that FA can be safely used as a nutraceutical adjunct, but they also suggest that ferulic acid alone may not dramatically lower blood glucose or HbA_1c_ in the short term in humans. To date, no large-scale clinical trial has specifically evaluated isolated ferulic acid for glycemic control in diabetic patients; thus, the efficacy of FA in lowering HbA_1c_ or improving insulin resistance in humans remains an open question.

### 2.4. Antihypertensive and Vascular Effects

#### 2.4.1. Evidence from Animal Studies

Ferulic acid (FA) has attracted interest for its potential benefits on blood pressure and vascular function. Epidemiological and clinical evidence suggests that diets rich in polyphenols, including hydroxycinnamic acids like ferulic acid, can help maintain vascular health and improve blood pressure control. As a major phenolic in whole grains, fruits, and vegetables, FA appears to contribute to these effects. In animal models of hypertension, ferulic acid consistently demonstrates antihypertensive and vasoprotective effects. For example, FA supplementation significantly lowers elevated blood pressure in spontaneously hypertensive rats and in diet-induced hypertensive models, while also improving measures of vascular function [23]. These improvements are attributed to FA’s impact on the arterial endothelium and oxidative balance. Mechanistic studies indicate that ferulic acid enhances endothelial nitric oxide (NO) bioavailability, promoting vasodilation, and reduces activation of the renin–angiotensin system, notably by suppressing angiotensin-converting enzyme (ACE) activity [23]. The net result is improved endothelial-dependent relaxation and attenuation of pathological vascular remodeling in hypertensive animals. FA’s potent antioxidant and anti-inflammatory properties also likely underlie these benefits: by scavenging reactive oxygen species and upregulating endogenous antioxidants, FA can protect endothelial cells from oxidative stress damage and preserve NO availability, thereby helping to normalize vascular tone [23,32,33]. Consistently, FA-treated rats show reduced vascular inflammation and stiffness alongside lower blood pressure [23].

Cell-based findings support these in vivo mechanisms. In isolated aortic segments and cultured vascular cells, FA has been shown to stimulate endothelial nitric oxide synthase and inhibit the production of vasoconstrictors, aligning with the enhanced vasorelaxation observed in vivo [23]. FA and its metabolites can directly induce vasodilation; for instance, the phase II metabolite ferulic acid-4-*O*-sulfate exerts significant vasodilatory effects in isolated arteries. FA also protects vascular endothelial cells from oxidative injury and inflammation, an effect noted in vitro by reduced expression of inflammatory cytokines and improved antioxidant enzyme activity [32]. These multi-modal actions, antioxidant, anti-inflammatory, and endothelial-protective, converge to improve overall vascular function in the context of hypertension.

#### 2.4.2. Evidence from Human Studies

Importantly, ferulic acid’s vascular benefits are not confined to preclinical studies. Emerging human data, while still limited, suggest that FA-rich diets or supplements can favorably influence surrogate markers of vascular health. In a randomized controlled trial, overweight individuals consuming a wheat-aleurone-rich diet (naturally high in ferulic acid) for eight weeks experienced a significant reduction in systemic oxidative stress, evidenced by a ~33% decrease in urinary 8-iso-prostaglandin F_2_α (an established marker of oxidative damage) compared to a refined wheat diet [26]. This improvement in oxidative stress is pertinent, as lower oxidative stress can translate to better endothelial function and vascular tone. Notably, the aleurone intervention did not significantly change blood pressure or inflammatory markers like C-reactive protein over the short duration, indicating that ferulic acid’s acute effects in humans may be subtler or require longer to manifest in overt hemodynamic changes. Similarly, polyphenol-rich food interventions (e.g., whole grains or coffees high in hydroxycinnamates) have demonstrated increases in circulating ferulic acid metabolites and enhanced antioxidant capacity without immediate changes in flow-mediated dilation or blood pressure in healthy adults. These findings underscore that, in humans, ferulic acid’s primary early effect may involve improving vascular oxidative stress and endothelial milieu, which are beneficial precursors to long-term vascular health, even if short-term blood pressure reductions are not pronounced.

It should be noted that direct clinical trials of isolated ferulic acid for hypertension are still scarce. One available human study (in hyperlipidemic patients) found that six weeks of high-dose ferulic acid supplementation (1000 mg/day) significantly improved lipid profiles and reduced oxidative stress and inflammation, which are cardiovascular risk factors, though blood pressure was not the primary endpoint [24]. While this lies just outside the last five-year window, it provides proof-of-concept that ferulic acid can be administered safely to humans and can modify cardiovascular risk markers. Overall, current human evidence, largely from dietary polyphenol interventions, suggests that ferulic acid confers vascular benefits mainly by mitigating oxidative stress and enhancing endothelial function, rather than acutely lowering blood pressure. Improvements in artery function (such as better endothelial responsiveness) may require longer-term intake or higher-risk populations to become evident.

Another translational aspect is the clinical use of sodium ferulate, a salt form of ferulic acid used in some countries (e.g., China) as a vasoprotective therapy. Sodium ferulate has been reported to improve endothelial function, inhibit platelet aggregation, and prevent thrombosis in cardiovascular patients [32]. Its efficacy in reducing microvascular complications and improving circulatory health in practice lends support to the idea that ferulic acid derivatives can exert tangible vascular benefits in humans. This real-world application complements the findings from controlled studies and highlights ferulic acid’s therapeutic potential against vascular dysfunction.

**Figure 2:**
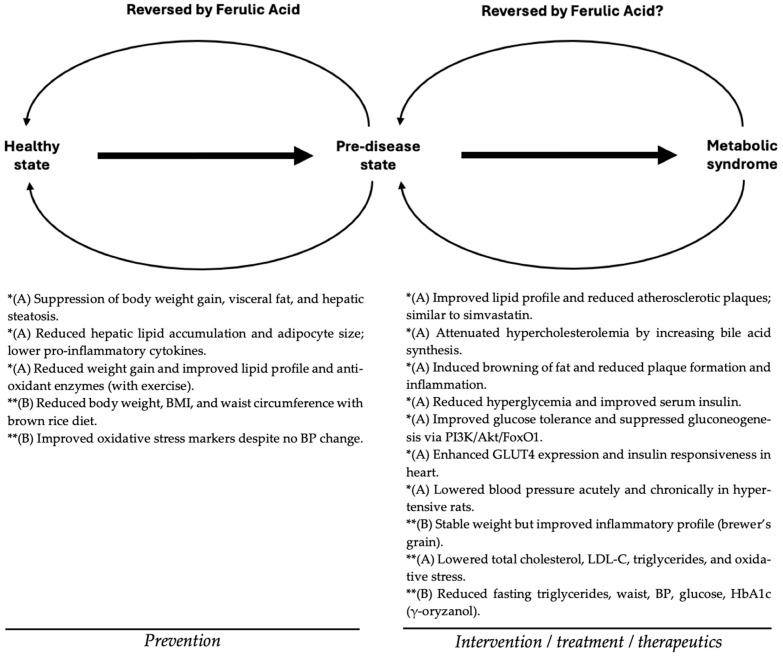
Proposed model for preventive and therapeutic effects of ferulic acid (A) or ferulic-acid-rich foods and supplements (B) against metabolic syndrome based on in vivo studies (*) and clinical studies (**).

Figure 2 summarizes the preventive and therapeutic effects of FA and FA-rich foods and supplements against metabolic syndrome, based on the in vivo and clinical evidence discussed in this review. According to these studies, FA can act at multiple stages of disease progression. In the context of prevention, FA may halt or reverse the early metabolic disturbances associated with a pre-disease state, contributing to the maintenance or restoration of a healthy metabolic profile. On the other hand, FA has also shown therapeutic potential by improving key biomarkers and symptoms associated with established metabolic syndrome, including obesity, dyslipidemia, hyperglycemia, hypertension, and systemic inflammation. This preventive/therapeutic model has previously been applied to dietary phenolics such as chlorogenic acid [34] and to food juices [35,36].

As shown in the left panel of Figure 2, most prevention-oriented effects have been demonstrated in animal studies using pure ferulic acid, with outcomes such as reduced weight gain, lower visceral adiposity, enhanced antioxidant enzyme activity, and improved lipid profiles. A few clinical studies using FA-rich foods, such as brown rice or wheat aleurone, have also reported preventive benefits, including reductions in waist circumference and oxidative stress markers. In contrast, the right panel of the figure highlights therapeutic findings observed in both in vivo and clinical studies. In animal models, pure FA has improved glycemic control through hepatic modulation of gluconeogenesis and insulin signaling pathways, enhanced lipid metabolism, and reduced atherosclerotic damage. Clinical studies using either pure FA or FA-rich food matrices have reported improvements in blood lipid profiles, inflammatory biomarkers, and glycemic parameters such as HbA_1c_.

Overall, while much of the mechanistic and preclinical evidence supports a role for pure ferulic acid in both the prevention and treatment of metabolic syndrome, the clinical data remain limited, especially for the isolated compound. Future human trials are necessary to determine the specific contribution of FA versus other components in FA-rich foods, and to validate its efficacy as a standalone nutraceutical or adjunctive therapeutic strategy.

## 3. Mechanisms of Action of Ferulic Acid in Metabolic Syndrome

**Figure 3:**
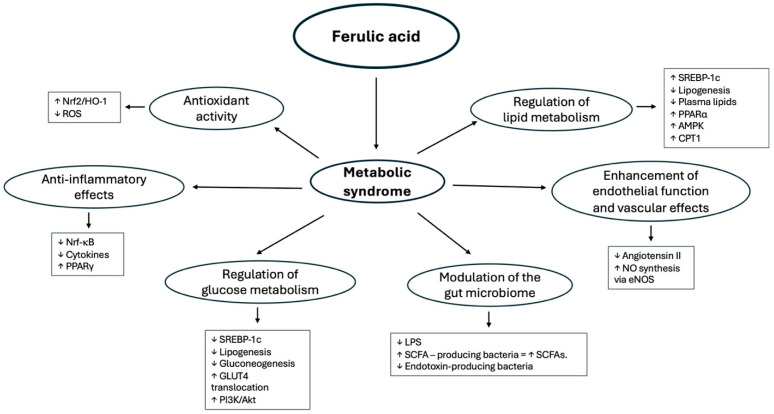
Mechanisms of action of ferulic acid in the amelioration of metabolic syndrome. Ferulic acid (FA) exerts protective effects against metabolic syndrome through multiple interrelated mechanisms, including enhancement of antioxidant defenses (↑ Nrf2/HO-1, ↓ ROS), suppression of chronic inflammation (↓ NF-κB, ↓ proinflammatory cytokines, ↑ PPARγ), improvement of glucose metabolism (↓ gluconeogenesis, ↑ GLUT4 translocation, ↑ PI3K/Akt), regulation of lipid metabolism (↓ SREBP-1c, ↑ PPARα, ↑ AMPK, ↑ CPT1), enhancement of endothelial function (↑ NO synthesis, ↓ angiotensin II signaling), and modulation of the gut microbiota (↑ SCFA-producing bacteria, ↓ endotoxin-producing bacteria, ↓ LPS). These coordinated effects collectively reduce insulin resistance, hyperlipidemia, inflammation, and vascular dysfunction associated with metabolic syndrome. Abbreviations: AMPK = AMP-activated protein kinase; CPT1 = carnitine palmitoyltransferase 1; eNOS = endothelial nitric oxide synthase; GLUT4 = glucose transporter type 4; HO-1 = heme oxygenase-1; LPS = lipopolysaccharide; NF-κB = nuclear factor kappa B; NO = nitric oxide; Nrf2 = nuclear factor erythroid 2–related factor 2; PI3K/Akt = phosphoinositide 3-kinase/protein kinase B pathway; PPARα/γ = peroxisome proliferator-activated receptor alpha/gamma; ROS = reactive oxygen species; SCFA = short-chain fatty acid; SREBP-1c = sterol regulatory element-binding protein 1c.

Ferulic acid (FA) is a plant-derived phenolic acid that exerts multifaceted biochemical effects to counteract the pathophysiology of metabolic syndrome (MetS). Recent studies demonstrate that FA’s health benefits stem from its potent antioxidant and anti-inflammatory activities, as well as its ability to favorably modulate glucose and lipid metabolism, improve endothelial function, and even alter the gut microbiome [15,23]. These mechanisms act in concert to ameliorate the hallmarks of MetS, including insulin resistance, dyslipidemia, hypertension, and obesity-related inflammation. Figure 3 provides a graphical overview of the molecular targets and pathways through which FA intervenes in MetS.

### 3.1. Antioxidant Activity

Oxidative stress is a key driver of insulin resistance and vascular dysfunction in MetS, and FA powerfully combats this through multiple antioxidant mechanisms. Chemically, the phenolic nucleus of FA directly scavenges free radicals, quenching reactive oxygen and nitrogen species (ROS/RNS) [37]. In vivo, FA bolsters endogenous antioxidant defenses by inducing cytoprotective enzymes. Treatment with FA has been shown to activate the Nrf2 pathway, leading to upregulation of heme oxygenase-1 (HO-1), glutathione S-transferase, superoxide dismutase, catalase, and other antioxidant enzymes [23]. For example, in diabetic animal models, FA supplementation significantly elevated hepatic and cardiac HO-1 expression and antioxidant enzyme activities, effectively restoring redox balance [23]. By reducing ROS accumulation, FA helps preserve mitochondrial function and ATP production in the liver and other tissues [7], thereby breaking the cycle of oxidative damage in MetS. The net result is that FA’s antioxidant action protects cells from oxidative injury and prevents ROS-induced activation of stress pathways implicated in metabolic syndrome progression.

### 3.2. Anti-Inflammatory Effects

Chronic low-grade inflammation underpins many MetS complications, and ferulic acid exerts broad anti-inflammatory effects to alleviate this burden. FA directly suppresses pro-inflammatory signaling pathways such as NF-κB and MAPK, thereby reducing the expression of cytokines and mediators [38]. In cultured macrophages, FA treatment inhibits LPS-induced iNOS and COX-2 expression and markedly reduces the release of inflammatory cytokines like TNF-α, IL-6, and prostaglandin E_2_ [38]. In one study, FA prevented the production of macrophage inflammatory protein-2 in virus-stimulated macrophages, highlighting its ability to restrain immune cell activation [38]. Animal models corroborate these findings: FA administration in rodents blunted tissue NF-κB activation and lowered levels of TNF-α and other cytokines elevated by a high-fat or high-carbohydrate diet [23]. Beyond inhibiting classic inflammatory pathways, FA also interferes with the NLRP3 inflammasome, a key driver of metabolic inflammation. For instance, FA was shown to block NLRP3 activation and IL-1β release in diabetic mice, an effect linked to improved insulin sensitivity and reduced tissue inflammation [23]. Additionally, FA can act as a ligand for PPARγ, a nuclear receptor with anti-inflammatory functions. By upregulating PPARγ expression and activity, FA further dampens NF-κB signaling and promotes an anti-inflammatory gene profile [39].

### 3.3. Regulation of Glucose Metabolism

Ferulic acid has demonstrated significant anti-diabetic effects by improving glucose homeostasis and insulin responsiveness. In dietary-induced MetS models, FA supplementation lowers fasting blood glucose and improves glucose tolerance [30]. Mechanistically, FA enhances insulin signaling in insulin-sensitive tissues. In vitro studies using palmitate-treated hepatocytes showed that FA activates the insulin/IGF-1 receptor–PI3K–Akt pathway, thereby promoting downstream glucose uptake and utilization [5,6]. FA increases membrane GLUT4 translocation and boosts insulin’s metabolic actions. Concurrently, FA suppresses hepatic gluconeogenesis. FA-treated hepatocytes and animals exhibit downregulated expression of key gluconeogenic enzymes G6Pase and PEPCK [5,6]. In a recent study, FA dose-dependently inhibited both enzymes, reducing glucose output from hepatocytes [5,6]. Additionally, FA increases glucokinase activity and glycogen synthase, promoting hepatic glycogen storage [23]. FA improves insulin sensitivity in vivo, with lower serum insulin and leptin and higher adiponectin levels in treated mice [30]. FA’s activation of AMPK in metabolic tissues likely contributes to these effects [7].

### 3.4. Regulation of Lipid Metabolism

FA treatment leads to consistent reductions in serum TG, total cholesterol, and LDL levels in animal models [15,37]. In high-fat-fed rats, dietary FA diminished hepatic lipid accumulation and plasma lipids [30]. At the molecular level, FA suppresses lipogenesis and promotes lipid oxidation. It downregulates SREBP-1c, FAS, and ACC in the liver [5,23], while activating AMPKα and inactivating ACC [7]. FA also increases CPT-1 expression and may activate PPARα, enhancing β-oxidation [7]. FA further aids cholesterol metabolism by inhibiting HMG-CoA reductase and upregulating hepatic LDL receptors [23]. These combined effects lower lipogenesis, promote fatty acid oxidation, and reduce plasma and hepatic lipid burden.

### 3.5. Enhancement of Endothelial Function and Vascular Effects

FA improves vascular endothelial function via several mechanisms. It enhances NO bioavailability by scavenging ROS and preserving NO from degradation [37]. In SHRs, FA restored acetylcholine-induced vasodilation and raised NO metabolites in plasma [37]. It also upregulates eNOS expression and activity [37]. Moreover, FA inhibits ACE activity, reducing Ang II formation and attenuating vasoconstriction [37]. In models of hypertension, FA supplementation lowered systolic blood pressure, reduced vascular remodeling, and improved arterial compliance [37].

### 3.6. Modulation of the Gut Microbiome

Ferulic acid modulates gut microbiota, contributing to its systemic metabolic effects. In high-fat-diet-fed mice, FA shifted the microbial composition, increasing SCFA-producing genera and decreasing endotoxin-producing bacteria [28]. This resulted in higher colonic SCFA levels, improved barrier function (tight junction expression), and reduced circulating LPS [28]. In ApoE^−/−^ mice, FA altered gut microbes and fecal metabolites, which was linked to lower SREBP-1 expression and hepatic lipogenesis [15]. These microbiota-mediated actions complement FA’s direct antioxidant, anti-inflammatory, and metabolic benefits.

## 4. Ferulic Acid as a Food Additive: Antimicrobial, Antioxidant, and Prebiotic Properties

In addition to its health-promoting effects as a nutraceutical, ferulic acid (FA) has gained considerable attention as a multifunctional food additive. Due to its broad-spectrum antimicrobial activity, potent antioxidant capacity, and emerging role in modulating the gut microbiome, FA has been studied in a variety of food matrices and model systems. These functionalities make it a promising natural preservative for extending shelf life, enhancing the stability of sensitive nutrients and bioactives and promoting gut health. Table 3 summarizes key in vitro, in vivo, and food application studies reporting the antimicrobial, antioxidant, and prebiotic effects of FA across diverse systems, highlighting its versatility and potential for clean-label food preservation and functional food development.

### 4.1. Antimicrobial Activity

Ferulic acid (FA) exhibits broad-spectrum antimicrobial effects against foodborne pathogens, including Gram-positive and Gram-negative bacteria. Reported minimum inhibitory concentrations (MICs) for FA are typically in the low milligram per milliliter range (on the order of 1–2 mg/mL) for organisms like *Listeria monocytogenes*, *Escherichia coli*, *Staphylococcus aureus*, and *Cronobacter sakazakii* [53]. The antimicrobial mechanism of FA is multifaceted. As a weak phenolic acid, undissociated FA can traverse microbial cell membranes and acidify the cytoplasm, disrupting enzyme activity and nutrient transport; concurrently, its phenolic hydroxyl group can insert into lipid bilayers and increase membrane permeability [53]. Indeed, phenolic acids tend to be most potent in their protonated form, and structural features of FA such as its 4-hydroxyl and 3-methoxy substituents enhance its efficacy (relative to simpler analogues like *p*-coumaric or caffeic acid) [53]. Adding hydrophobic moieties (e.g., alkyl esters) can further boost the antimicrobial potency of ferulate derivatives [53]. These modes of action, membrane disruption and intracellular acidification, result in growth inhibition and cell injury in various pathogens. Notably, FA’s activity is generally stronger against Gram-positive bacteria (which lack an outer membrane), but it can also inhibit Gram-negative bacteria at sufficient concentrations [53].

In culture media, FA has demonstrated bacteriostatic or bactericidal effects on priority food pathogens such as *L. monocytogenes* (MIC ~2.0 mg/mL) and *E. coli* (MIC ~1.5 mg/mL) [53]. More importantly, there is growing evidence of FA’s efficacy in real food systems and its potential to be used in hurdle approaches. For example, in a cold-cut meat model system, the combination of ferulic acid with another phenolic acid (caffeic acid) showed a synergistic inhibitory effect. When used at 150–200 ppm each, the ferulic + caffeic acid combination produced greater bacterial reductions than either acid alone, achieving up to a 3.6 log CFU/g reduction of *E. coli O157:H7* on meat after 72 h at 4 °C [38]. This synergistic effect was evident both in broth and in meat, indicating that combined natural preservatives can overcome some of the efficacy losses seen in foods (where antimicrobials often bind to food matrices). In the same study, ferulic and caffeic acids also inhibited *L. monocytogenes* in meat, and overall, the authors concluded that these phenolics, individually and especially together, can improve the safety of ready-to-eat meats (cold cuts) by reducing pathogen loads [38]. Ferulic acid also interacts synergistically with certain natural antimicrobial peptides. For instance, combining ferulic acid with ε-polylysine (a cationic biopreservative) dramatically enhances the antimicrobial efficacy against the spoilage bacterium *Shewanella putrefaciens*. Cui et al. [39] found that using only 1/4 of the MIC of FA together with 1/4 MIC of ε-polylysine completely inhibited *S. putrefaciens* growth, whereas each agent alone at that dose was ineffective. This FA + ε-polylysine combination (FICI ≤ 0.5) damaged the bacterial cell structure, as evidenced by increased cell membrane permeability and leakage of intracellular contents. Such synergism likely arises from ε-polylysine disrupting the outer cell envelope and facilitating FA’s entry and action on internal targets.

FA’s antimicrobial action has been demonstrated in various food applications. In fresh produce and juices, FA can help control pathogens without thermal treatment. As an example, addition of 1500 mg/L FA to fresh apple and orange juices significantly inhibited the growth of *L. monocytogenes* during refrigerated storage [40]. After nine days at 4 °C, FA-fortified juices had a lower *Listeria* population compared to untreated juice, while indigenous juice microbiota were largely unaffected (meaning FA targeted the inoculated pathogen without disturbing native flora) [40]. The FA fortification did cause some changes in juice properties (e.g., increased phenolic content, higher antioxidant capacity, and slight acidification and color darkening). Sensory scores of the FA-treated juices were somewhat lower, indicating a need to optimize concentration for minimal sensory impact [40]. Nonetheless, this study highlights FA’s promise as a natural antimicrobial for minimally processed juices, especially against acid-tolerant pathogens like *L. monocytogenes*.

In dairy foods, ferulic acid has also shown efficacy against *Listeria*. In high-moisture, neutral-pH fresh cheeses (e.g., Hispanic-style queso fresco), FA can inhibit Listeria and even prevent the development of resistant subpopulations. Research has demonstrated that incorporating FA into fresh cheese, either alone or in combination with the bacteriocin nisin, suppresses *Listeria* growth during refrigerated storage [41]. Notably, *Listeria* exposed to sublethal FA did not develop increased resistance after repeated passages [41], an encouraging finding that suggests ferulic acid may not readily trigger adaptive tolerance mechanisms. This makes FA an attractive adjunct hurdle in cheeses, potentially used alongside nisin to broaden the antimicrobial spectrum and improve the safety of products prone to *Listeria* contamination (e.g., soft cheeses).

In bakery products, ferulic acid’s direct antimicrobial role is less documented than its antioxidant role (see below), but as a natural phenolic, it could inhibit molds or bacterial spoilers to some extent. Its practical use in bread or cake would likely be in combination with other preservatives, given that baking conditions and high pH may reduce its activity [54]. Finally, ferulic acid is being explored in edible films and coatings for its antimicrobial benefits. A recent study incorporated FA into a konjac glucomannan-based edible coating alongside ε-polylysine and found that this composite film markedly improved the microbial quality of refrigerated seafood. Specifically, sea bass fillets coated with a KGM/ε-PL/ferulic acid film had significantly lower total viable counts during 4 °C storage, compared to control or films without FA [42]. The FA-infused coating also inhibited the growth of spoilage bacteria like *S. putrefaciens*, delaying fish spoilage and extending shelf life [42]. Ferulic acid’s ability to remain active in the film matrix and diffuse to the food surface where microbes reside is key to this application.

Notably, unlike some highly hydrophobic essential oil constituents (e.g., eugenol), which can partition into fatty phases and lose efficacy in emulsified or high-fat foods, ferulic acid’s relatively higher water solubility allows it to retain antimicrobial activity in complex food systems [43,55]. Pernin et al. [43] observed that in an oil-in-water emulsion, ferulic acid continued to inhibit *L. monocytogenes* effectively, whereas eugenol (which is more lipophilic) became sequestered in the oil phase and had reduced activity. This suggests that ferulic acid can be a useful antimicrobial in foods like sauces, dressings, or emulsified meats, either alone or in combination with other hurdles, to ensure pathogen control even in heterogeneous matrices.

### 4.2. Antioxidant Properties

One of the most well-recognized functions of ferulic acid as a food additive is its potent antioxidant activity. Chemically, FA is a phenolic acid with a conjugated aromatic ring and unsaturated side chain that enable it to donate hydrogen atoms (or electrons) to quench free radicals. Upon donating a hydrogen, FA forms a resonance-stabilized phenoxy radical, which is relatively unreactive [56]. In essence, FA can terminate radical chain reactions that lead to lipid oxidation. Its effectiveness as a natural antioxidant has been demonstrated in a variety of food systems, often matching or exceeding the performance of synthetic antioxidants in research settings. For example, in muscle foods and emulsions, FA inhibits the formation of lipid oxidation products such as peroxides and aldehydes. In situ trials in a dried meat product showed that 0.1% FA significantly suppressed oxidative rancidity over a six-month storage period, keeping thiobarbituric acid reactive substances (TBARS, a measure of malondialdehyde from fat oxidation) below the threshold of sensory detection of rancidity [44]. Hernández-Jaime et al. [44] found that dried beef snacks formulated with ferulic acid had TBARS values well under those of control samples, and panelists could not distinguish any off-flavors, indicating effective prevention of lipid oxidation by the added FA. In the same study, ferulic acid also maintained a higher antioxidant capacity (measured by DPPH, FRAP, and ABTS assays) in the meat during storage, reflecting its ability to scavenge free radicals and protect the meat’s fatty components. Notably, FA-treated dried meat showed no more oxidation at six months than control samples did at two months, illustrating how ferulic acid prolonged the product’s oxidative shelf life [44].

Ferulic acid has also proven effective in bulk oils and oil-in-water systems. Its addition to plant oils rich in polyunsaturated fatty acids can dramatically enhance oxidative stability. For instance, in cold-pressed flaxseed oil (high in omega-3 linolenic acid), the induction time for oxidation (measured by Rancimat) increased linearly with FA concentration [45]. Even at modest levels (e.g., 0.02–0.1%), FA delayed the onset of peroxide formation, thereby extending the oil’s shelf life compared to an untreated control. Similarly, encapsulated fish oil (an omega-3 concentrate prone to oxidation) benefited from ferulic acid incorporation: FA added into the wall matrix of nanofiber encapsulates significantly slowed lipid peroxidation during storage [46]. Importantly, the FA did not adversely affect the oil’s release or sensory properties. These results suggest that ferulic acid can protect highly unsaturated oils (like omega-3 fish oils) from rancidity, making it valuable for fortification of foods with fish oil or algae oil (which tend to develop fishy off-odors upon oxidation). In frying oils or repeated-use oils, ferulic acid (or its more lipophilic derivatives) could similarly inhibit oxidation and polymerization, though further studies are needed in high-temperature conditions.

In addition to suppressing primary lipid oxidation (peroxide formation), FA also reduces secondary oxidation products and other markers of quality degradation. For example, FA addition in meat and fish systems correlates with lower values of total volatile base nitrogen (TVB-N) and inhibited formation of off-flavor volatiles [39,42]. In FA-treated crayfish tails stored at 4 °C, the increase in TVB-N (a measure of breakdown of proteins and amines during spoilage) was significantly slower than in controls, consistent with FA’s antioxidant and antimicrobial actions delaying spoilage [39]. The same study noted that ferulic acid (especially when combined with ε-PL) curtailed the production of malodorous compounds such as trimethylamine and certain aldehydes responsible for “fishy” smell [42]. This highlights how FA’s antioxidant property contributes not only to nutritional quality preservation but also to maintaining desirable flavor and aroma in foods by preventing oxidative off-notes.

A notable aspect of ferulic acid’s antioxidant function is its synergistic interaction with other antioxidants. FA is often termed a “network antioxidant” because it can regenerate or potentiate classic antioxidants like vitamins C and E. In fact, ferulic acid in combination with ascorbic acid (vitamin C) and α-tocopherol (vitamin E) exhibits a famously synergistic effect. Trombino et al. [47] demonstrated that FA was the most effective single antioxidant among those tested in a liposomal membrane system, and when used in combination with α-tocopherol or ascorbate, it showed synergistic protection of lipids from oxidation. The mechanism involves ferulic acid stabilizing the tocopheroxyl and ascorbyl radicals (which form when vitamins E and C scavenge radicals), thereby regenerating the active antioxidant form of the vitamins. In practical terms, adding a small amount of ferulic acid can double the efficacy of a vitamin C + E antioxidant system. This was illustrated in a formulation where 0.5% FA was added to a solution of 15% L-ascorbic acid and 1% α-tocopherol: the mixture’s overall antioxidant capacity and photostability were significantly enhanced compared to the vitamins alone [57]. In foods, this means ferulic acid can be used alongside traditional antioxidants like tocopherols (e.g., mixed tocopherols from plant oils) and ascorbates to achieve a greater protective effect with lower doses of each. For instance, in an oil-in-water emulsion, an antioxidant cocktail of FA + ascorbyl palmitate + tocopherol might suppress lipid oxidation far better than any single component, due to such synergistic recycling of radicals. This strategy is appealing for “clean-label” product development, as it relies on naturally occurring antioxidants working in concert.

Ferulic acid’s antioxidant utility extends to protecting other sensitive food constituents as well. It can guard the stability of pigments, vitamins, and bioactive compounds that are prone to oxidation. For example, fruit and vegetable colors based on anthocyanins can be stabilized by ferulic acid through copigmentation (see next section), and vitamins like vitamin C can be preserved in the presence of FA. In one study with fresh juices fortified with FA, not only was microbial growth controlled, but the endogenous vitamin C and phenolic content were better retained during storage, likely because FA prevented oxidative degradation of these nutrients [40]. Similarly, ferulic acid may help stabilize carotenoids and other easily oxidizable phytonutrients in functional foods. Overall, the incorporation of ferulic acid as a natural antioxidant in emulsions, meat products, frying oils, and packaging materials has shown substantial reductions in oxidation indicators such as peroxide value, TBARS, conjugated dienes, and hexanal content [44,58]. By mitigating lipid peroxidation, ferulic acid helps maintain food quality (flavor, color, nutritional value) and extends shelf life, satisfying the demand for effective “clean-label” antioxidants in the food industry.

### 4.3. Stabilization of Bioactives

Beyond its direct antioxidant action, ferulic acid contributes to the stabilization of various bioactive compounds in foods, ranging from pigments to vitamins to flavor molecules. One important phenomenon is copigmentation, wherein ferulic acid non-covalently associates with anthocyanin pigments to form stable, colored complexes. Anthocyanins (the red-blue pigments in berries, grapes, purple corn, etc.) are known to be unstable to pH changes, light, and heat, often leading to color fading or browning in processed foods. Ferulic acid, as a copigment, can stack planar to the anthocyanin’s flavylium ring via π–π interactions, thereby protecting the chromophore from hydration or nucleophilic attack (which cause pigment degradation) [59]. The result of copigmentation is an immediate hyperchromic (color-deepening) effect and improved color retention over time. For instance, Pangestu et al. [11] showed that adding ferulic acid or chlorogenic acid to model beverages colored with elderberry and purple carrot anthocyanins intensified the color and slowed pigment loss during eight weeks of storage. Beverages with ferulic acid had higher initial chroma (color saturation) and exhibited a smaller decline in anthocyanin concentration over time compared to non-copigmented controls. In elderberry pigment solutions, ferulic acid increased the half-life of color (measured as chroma retention), helping to maintain a vivid hue even as some anthocyanin degradation occurred [48]. Likewise, copigmentation of anthocyanins from blackcurrant with ferulic acid was found to extend their thermal stability at moderate pH (around 6.0), effectively increasing the pigment half-life under heat stress [48].

Intermolecular copigmentation with ferulic acid has also been reported to significantly improve the heat stability of *Hibiscus sabdariffa* anthocyanins, which translated into better color retention in high-temperature applications [49]. These findings are valuable for the development of natural colorants: by formulating anthocyanin-rich extracts together with ferulic acid (or derivatives), food manufacturers can achieve more intense and long-lasting colors in products like beverages, yogurts, confections, and baked goods, reducing color fading during shelf life. Notably, ferulic acid is especially effective as a copigment for anthocyanins acylated with similar hydroxycinnamic acids (e.g., ferulic or *p*-coumaric acylated anthocyanins), as these have an innate affinity for stacking interactions [60].

Ferulic acid also helps stabilize essential vitamins such as vitamin C (ascorbic acid) and vitamin E (tocopherols) in fortified foods. Both vitamins are prone to oxidation: ascorbic acid can oxidize to dehydroascorbic acid, and α-tocopherol can oxidize to tocopheroxyl radicals or quinones, especially in the presence of free radicals or metal catalysts. By scavenging initiating radicals and chelating trace metals, ferulic acid shields these vitamins from oxidative loss. Furthermore, as mentioned earlier, FA can regenerate oxidized ascorbate and tocopherol, essentially recycling these vitamins within the food matrix [47]. This has practical implications: in an enriched beverage or an emulsion containing vitamins C and E, inclusion of ferulic acid can double the protection against vitamin degradation [47,57]. Empirically, beverages or model systems containing ferulic acid show higher retained ascorbate content after storage compared to those without FA, under identical conditions (by preventing ascorbate oxidation to nondetectable forms). Thus, ferulic acid helps ensure that the added nutritional benefits of vitamins are not lost during processing or storage.

Another category of bioactives stabilized by ferulic acid are polyunsaturated fatty acids, particularly omega-3 fatty acids (like EPA and DHA). While not a “bioactive” in the classical phytochemical sense, omega-3s are nutritionally valuable but chemically fragile (highly susceptible to peroxidation). Ferulic acid’s role here is as an antioxidant protector: as described in the section on antioxidants, FA can substantially delay omega-3 oxidation in oils and emulsions [45]. In one approach, researchers have synthesized amphiphilic derivatives (e.g., lipophilized ferulates) to better integrate into oil phases; these compounds (such as lauryl ferulate or lipoic ferulate) showed even stronger efficacy in stabilizing fish oil than FA itself, outperforming conventional antioxidants like BHT in some cases [61]. For functional foods enriched with omega-3 (e.g., omega-3 eggs, breads, or milk), ferulic acid or its derivatives could thus protect the fatty acids from oxidation, preserving their nutritional quality and preventing fishy odors.

Ferulic acid may also interact with proteins and enzymes, contributing to their stability. In plant systems, FA is known to cross-link polysaccharides and proteins (it naturally cross-links arabinoxylans in cereal cell walls via oxidative coupling). In a food context, ferulic acid can undergo enzymatic or radical-mediated cross-linking with proteins such as gelatin, whey protein, or soy protein, which has been exploited to strengthen edible films and gels. While this is a different mode (a covalent interaction rather than simple protection), it does stabilize the protein network by forming ferulate bridges. For instance, ferulic acid has been used to cross-link soy protein isolates in films, improving their tensile properties and water resistance [50]. Additionally, by scavenging radicals, FA can prevent protein oxidation (e.g., carbonyl formation) in foods, thereby maintaining protein functionality (solubility, emulsifying properties) and nutritional value. In muscle foods, oxidation can lead to protein aggregation and toughness; ferulic acid’s antioxidant action indirectly preserves textural qualities by limiting such oxidative protein changes [44].

Flavor and aroma compounds in foods are another class that benefits from ferulic acid’s stabilizing presence. Many key aroma molecules (ethers, terpenes, unsaturated aldehydes, etc.) are sensitive to oxidation or polymerization, which can dull flavor profiles during storage. By curbing oxidative reactions, ferulic acid helps retain the volatile flavor fraction. A striking demonstration was in the refrigerated fish fillet study: the fillets coated with ferulic acid had significantly lower levels of volatile amine and ketone compounds associated with fish spoilage odors [42]. Essentially, FA prevented the oxidative breakdown of trimethylamine N-oxide (found in fish muscle) into malodorous trimethylamine, as well as inhibiting fatty acid oxidation, which produces rancid-smelling aldehydes [42]. The FA-coated fish were judged to have a fresher flavor for longer, attesting to FA’s aroma-protective effect. Similarly, in baked goods, ferulic acid (especially from natural sources like rice bran or wheat bran extracts) can protect flavor by delaying lipid oxidation, which would generate off-flavors. It might also help stabilize certain aroma compounds that are antioxidants themselves (e.g., vanillin is structurally similar to ferulic and is relatively stable in its presence).

### 4.4. Prebiotic Potential

Beyond its preservative functionalities, ferulic acid may impart health benefits as a prebiotic or modulator of the gut microbiota. In this section, the prebiotic properties of FA are discussed in a scientific context, focusing on its effects on specific microbial taxa, short-chain fatty acid production, and gut barrier function. Prebiotics are substrates that are utilized by host microorganisms, conferring a health benefit, and while classical prebiotics are typically fibers or oligosaccharides, certain polyphenols like ferulic acid also exhibit prebiotic-like effects (by influencing microbial composition and metabolism in the colon). Ferulic acid in the diet largely comes from plant cell wall components (such as arabinoxylan fiber in cereal brans), where it exists in bound form (ferulate esters). In the human gut, commensal bacteria, particularly some *Bifidobacterium* and *Lactobacillus* species, produce ferulic acid esterases that can release ferulic acid from fiber, allowing it to be metabolized by the gut microbiota [62]. The presence of fermentable ferulic acid and its derivatives can selectively stimulate beneficial microbes. Studies have shown that when ferulic acid or feruloylated fibers are consumed, there is an increase in populations of certain saccharolytic and butyrogenic bacteria. For instance, *Bifidobacterium* spp., which are considered beneficial probiotics, can utilize ferulic acid and often increase in abundance in ferulic-supplemented diets [63]. Likewise, ferulic acid has been reported to enrich butyrate-producing bacteria in the gut, such as members of *Clostridiales* Cluster IV/XIVa (e.g., *Faecalibacterium prausnitzii* or *Roseburia* spp.), which ferment ferulic and other phenolics to short-chain fatty acids (SCFAs) like butyrate [28]. Butyrate is a key SCFA that nourishes colonocytes, strengthens the intestinal barrier, and exerts anti-inflammatory effects in the gut. In a 12-week study on high-fat-diet-induced obese mice, oral administration of ferulic acid (100 mg/kg) significantly altered the gut microbiota composition in favor of SCFA-producing taxa and increased the concentrations of total SCFAs (especially butyrate) in the colon [28]. The FA-treated mice showed higher abundances of genera like *Olsenella*, *Eisenbergiella*, *Faecalibaculum*, and other butyrate producers, alongside a reduction in opportunistic endotoxin-producing Gram-negative bacteria [28]. Correspondingly, these mice had improved markers of gut health: enhanced expression of tight junction proteins and mucins (indicating a stronger intestinal barrier), reduced colonic inflammation, and lower systemic endotoxin (LPS) levels [28]. The study concluded that ferulic acid mitigated gut barrier dysfunction and inflammation by reshaping the microbiota towards a more favorable, SCFA-rich profile [28].

One important aspect of ferulic acid’s microbial metabolism is the production of bioactive metabolites. Gut bacteria can break down ferulic acid into various metabolites, including phenolic acids (like vanillic acid, caffeic acid), phenylpropionic acids, and SCFAs (acetate, propionate, and butyrate) [62]. Some of these metabolites have their own bioactivities. For example, dihydroferulic acid (formed by microbial hydrogenation of the side chain) and ferulic acid itself can be absorbed and exert antioxidant and anti-inflammatory effects systemically. Butyrate, as mentioned, has local anti-inflammatory effects in the gut and can signal through GPR43/GPR109A receptors to regulate immune function [28]. Propionate can influence gluconeogenesis and satiety via the gut–liver axis. In essence, the gut microbiota’s fermentation of ferulic acid yields compounds that contribute to metabolic health. In vitro fecal fermentation studies have observed that ferulic acid stimulates butyrate production while suppressing proteolytic putrefaction compounds, suggesting a shift towards a healthier fermentation profile [62].

Another observed benefit of ferulic acid’s interaction with gut microbiota is the modulation of inflammation and oxidative stress in the gut. Ferulic acid can downregulate pro-inflammatory pathways (like NF-κB) in intestinal cells, either directly or via SCFA-mediated mechanisms. Tian et al. [28] noted that FA supplementation in mice led to inhibition of the colonic TLR4/NF-κB signaling cascade, the pathway often triggered by endotoxins, thereby reducing the production of inflammatory cytokines in the gut. This was attributed to both the reduced endotoxin load (thanks to fewer Gram-negative bacteria and a stronger mucus barrier) and possibly direct antioxidative, anti-inflammatory action of ferulic acid and its metabolites on gut epithelium. Improved gut barrier integrity was evidenced by higher tight junction protein levels in FA-treated mice, correlating with reduced intestinal permeability (and hence lower systemic inflammation) [28]. These findings align with the concept that ferulic acid mediates a beneficial crosstalk between diet, microbiota, and host. In other studies, ferulic acid has been shown to ameliorate markers of metabolic syndrome (such as insulin resistance and hepatic steatosis) partly by modulating gut microbiota composition [23,62]. It appears that ferulic acid increases the relative abundance of gut microbes associated with leanness and anti-inflammatory effects, including not only *Bifidobacterium* but also *Akkermansia muciniphila* in some reports (a mucus-degrading bacterium linked to improved metabolic health).

It is worth noting that much of ferulic acid’s prebiotic effect may occur when it is delivered as part of a fiber matrix (e.g., wheat bran). In that context, ferulic acid is slowly released in the colon, continuously feeding the microbiota. Pure ferulic acid can also reach the colon if not fully absorbed in the small intestine. It has limited bioavailability orally, with significant portions undergoing phase II conjugation or microbial metabolism. Unabsorbed FA and its conjugates thus become substrates for colonic microbes. Research suggests that about 40–50% of ingested ferulic acid (from a high-bran diet) can be recovered as microbial metabolites, indicating substantial fermentation. The net result is often an increase in total SCFA output. Indeed, a human trial with wheat bran (rich in bound ferulic acid) showed higher total fecal SCFA and a shift towards a greater proportion of butyrate in feces [51]. Two possible mechanisms were proposed: (1) ferulic acid release and fermentation acidify the colon, favoring butyrogenesis; (2) ferulate-utilizing bacteria cross-feed other commensals, enhancing butyrate production [51,52].

## 5. Ferulic Acid in Cosmetic Applications

In addition to its diverse health-promoting and preservative roles in foods, ferulic acid (FA) has gained increasing attention in the cosmetic and dermatological fields. Its multifunctional bioactivities, including photoprotection, anti-inflammatory effects, and stabilization of other antioxidants, have positioned FA as a key natural ingredient in cosmeceutical formulations. Table 4 summarizes preclinical and clinical studies investigating FA’s role in skin protection, repair, and anti-aging applications, highlighting its potential to enhance product efficacy while meeting consumer demand for safe and natural bioactives.

### 5.1. Photoprotection

Ferulic acid acts as a multitargeted photoprotective agent. Although ferulic acid alone cannot absorb sufficient UV radiation to be considered an effective filter, it can enhance the action of some UV filters through several mechanisms. It directly absorbs UV light in both the UVB and UVA range (absorption peaks around 284 and 307 nm), thereby reducing the amount of radiation penetrating the skin [72]. More importantly, FA serves as a potent antioxidant “shield” that neutralizes free radicals generated by UV exposure. This antioxidant capacity helps prevent oxidative damage to skin cells and biomolecules. In fact, FA is a highly effective scavenger of superoxide and peroxyl radicals, inhibiting lipid peroxidation in skin membranes. By quenching these reactive oxygen species, FA protects cellular DNA, proteins, and lipids from UV-induced oxidative stress [72].

In vivo studies and clinical trials have demonstrated tangible photoprotective benefits. When added to sunscreen formulations, even low concentrations of FA significantly boost the protection offered by conventional UV filters. Peres et al. [72] found that incorporating 1% FA into a broad-spectrum sunscreen raised the in vivo sun protection factor (SPF) by ~37% and the UVA protection factor by ~26% (versus the same sunscreen without FA). This indicates a synergistic effect between FA and UV filters, effectively broadening and strengthening the formulation’s defense against both UVB and UVA rays [72]. Notably, the FA-containing sunscreen also showed a capacity to reduce UV-induced skin inflammation: test subjects had less erythema (redness) after UV exposure compared to those using the UV filters alone. The good photostability and skin compatibility of FA in these formulations suggest it can be safely used to create “multifunctional” sunscreens that not only absorb UV but also deliver antioxidant and anti-inflammatory benefits.

Beyond sunscreens, ferulic acid is often combined with other antioxidants to enhance photoprotection. A classic example is its synergistic use with vitamins C and E. Multiple controlled trials have shown that a topical antioxidant serum containing ferulic acid with vitamins C (L-ascorbic acid) and E provides significant protection against UV damage in human skin [57,73]. In these studies, skin pre-treated with the vitamin C + E + ferulic serum experienced markedly less UVB-induced erythema and sunburn cell formation compared to untreated skin or skin treated with vehicle alone. The inclusion of ferulic acid was key: it helps stabilize vitamins C and E and doubles their photoprotective efficacy on skin [57]. Mechanistically, ferulic acid regenerates vitamin E from its oxidized form and protects vitamin C from rapid oxidation, prolonging the activity of both vitamins in the skin [85]. The result is a synergistic antioxidant network that can neutralize a broader range of reactive species generated by UV, providing an “enhanced SPF” effect even when no sunscreen is present [86]. Clinically, such combinations have been shown to significantly reduce acute UV-induced erythema by day 4 of UV exposure, compared to controls [74]. In summary, ferulic acid contributes to photoprotection through a three-fold approach: directly absorbing UV photons, scavenging free radicals before they can damage skin, and synergistically boosting the efficacy of other antioxidants and sunscreens in formulations.

### 5.2. Skin Repair and Anti-Inflammatory Effects

Beyond its preventive role against photoaging, ferulic acid also aids in skin repair and inflammation modulation. It has demonstrated significant wound-healing properties in preclinical models. FA can accelerate the closure of wounds in diabetic rats, likely by enhancing granulation tissue formation and collagen deposition while curbing excess inflammation [69]. At the cellular level, ferulic acid modulates key signaling pathways involved in the inflammatory response. For example, in a mouse model of atopic dermatitis (an inflammatory skin condition), topical ferulic acid markedly alleviated dermatitis symptoms, reducing redness, swelling, and epidermal thickening, by downregulating pro-inflammatory cytokines such as interleukin-6 and TNF-α [71]. The treated mice showed restoration of a healthier skin barrier and less immune cell infiltration, highlighting FA’s anti-inflammatory efficacy in vivo [71]. These findings align with in vitro studies on human skin cells: ferulic acid can suppress UV or chemical-induced upregulation of inflammatory mediators in keratinocytes and fibroblasts, partly via activation of Nrf2 antioxidant responses and inhibition of NF-κB pathways.

Clinically, ferulic acid has shown benefits as a skin-calming and post-procedure agent. Its anti-inflammatory action can soothe irritation and erythema following stressors like UV exposure or dermatological treatments. In one study, applying a ferulic acid antioxidant serum after laser resurfacing (ablative laser treatment) accelerated skin recovery: patients experienced improved skin texture and less post-inflammatory hyperpigmentation compared to untreated controls, with no increase in irritation [75]. Ferulic acid’s ability to neutralize oxidative stress in wounded or irritated skin likely underlies these benefits. There is also evidence that FA can mitigate UV-induced inflammation specifically. Sauce et al. [76] showed that a sunscreen formulation containing ferulic acid produced lower levels of UV-triggered inflammatory markers in skin ex vivo than an equivalent formulation without FA. In patch tests on human volunteers, the FA-enriched formulation significantly reduced skin erythema caused by an irritant (methyl nicotinate), demonstrating anti-inflammatory efficacy on acute contact inflammation [76].

Finally, ferulic acid may assist in skin barrier repair. A recent randomized controlled trial in patients with mild-to-moderate rosacea, a condition involving impaired barrier function and chronic inflammation, found that adding topical ferulic acid to standard therapy improved outcomes [77]. After six weeks, the ferulic-treated group showed significantly greater reduction in rosacea lesions (inflammation and pustules) and enhanced restoration of the skin’s barrier integrity (measured by transepidermal water loss and skin hydration) compared to controls. Patients receiving ferulic acid reported minimal irritation and faster relief of symptoms. These clinical results underscore that ferulic acid’s anti-inflammatory and antioxidant actions translate into visible improvements in skin healing and comfort. Whether for a sunburn, post-procedure erythema, or an inflammatory disorder, ferulic acid helps calm the skin, reduce redness, and promote recovery of the skin’s normal structure.

### 5.3. Skin Brightening

Ferulic acid also contributes to a more even skin tone and brightness through multiple mechanisms. Chief among these is its ability to inhibit melanin synthesis. FA is a competitive inhibitor of *tyrosinase*, the key enzyme in melanogenesis that catalyzes melanin production in melanocytes. In biochemical assays, ferulic acid directly binds to the active site of tyrosinase, reducing the enzyme’s activity and thus slowing the conversion of tyrosine to melanin pigment [64]. In cultured B16 melanoma cells, ferulic acid treatment significantly reduced melanin content without harming the cells, confirming a depigmenting effect via tyrosinase inhibition [64]. Notably, ferulic acid was more effective than the related cinnamic acid (caffeic acid) in this regard, which researchers attributed to ferulic’s stronger binding affinity for tyrosinase and its ability to also block tyrosinase phosphorylation by casein kinase 2. By hindering both the enzyme’s activity and its activation, ferulic acid can markedly suppress melanogenesis at the cellular level.

This biochemical property translates into visible skin-brightening benefits when ferulic acid is used over time. Topical products containing ferulic acid have shown efficacy in reducing hyperpigmentation, especially when used in combination with other actives. For example, Milani et al. [78] reported that a serum combining ferulic acid with an Antarctic plant extract and vitamin C led to a significant reduction in dark spots and a brighter skin complexion after four weeks of use (*p* < 0.05). In patients with melasma (a form of hyperpigmentation), ferulic acid has been used as part of combination peels or topical regimens to lighten pigmented patches. A split-face study by Mazurek and Pierzchała [79] compared two melasma treatments and noted that the side treated with a ferulic-acid-containing formula achieved greater decreases in melanin index and pigment intensity, although both sides saw improvements. Post-inflammatory hyperpigmentation (PIH), which occurs after acne or skin injury, may also be improved with ferulic acid. In a 2020 trial, women with facial hyperpigmentation who underwent Q-switched laser treatment had better pigmentation clearance when they used a daily antioxidant serum with 0.5% ferulic acid + vitamins C/E post-laser, compared to using no antioxidant [75]. The ferulic combination not only helped fade the laser-induced PIH more rapidly but also improved overall skin tone and texture in the treated areas.

Furthermore, ferulic acid’s photoprotective effects complement its brightening action. By neutralizing UV-induced oxidative stress and inhibiting UVA-induced melanogenesis in the skin, FA can help prevent new pigment formation triggered by sun exposure. One study noted that ferulic acid treatment protected human skin samples from UVB-induced increases in melanin, essentially blunting the skin’s tanning response [87]. While that is an older finding, recent research continues to support ferulic acid’s role in controlling pigmentation. The antioxidant network of C + E + ferulic acid, for instance, has been shown to reduce not just erythema but also the intensity of UV-induced pigmentation when applied before UV exposure [86]. Over several months of use, ferulic-acid-containing serums can gradually brighten areas of mottled pigmentation and even out the complexion. Its gentle mode of action (tyrosinase inhibition and antioxidant protection) makes ferulic acid a favorable brightening agent with a low risk of irritation or rebound hyperpigmentation. This is especially valuable for managing conditions like melasma or PIH, where inflammation can exacerbate pigment problems.

### 5.4. Anti-Aging and Anti-Photodamage

Ferulic acid is widely recognized for its anti-aging properties, particularly in preventing and reducing photodamage. A core aspect of skin aging (especially photoaging) is the degradation of dermal collagen and elastin fibers due to UV-induced oxidative stress and matrix metalloproteinases (MMPs). Ferulic acid helps protect these structural proteins and maintain skin firmness. It has been shown to inhibit UV-induced MMP expression, thereby slowing collagen breakdown. In a mouse study, topical ferulic acid significantly suppressed the increase of MMP-9 and MMP-2 that normally occurs after chronic UVB exposure, ultimately reducing collagen damage and wrinkle formation [70]. Similarly, in vitro work with human dermal fibroblasts found that ferulic acid pre-treatment protected the cells from UVA radiation—fibroblasts showed higher viability and less collagenase activity after UVA when ferulic acid was present [65]. By attenuating the UV-activated pathways that lead to collagen breakdown, ferulic acid preserves the skin’s extracellular matrix, which translates to fewer wrinkles and improved elasticity over time.

The antioxidant capacity of FA also means it mitigates other aging mechanisms, such as protein oxidation and lipid peroxidation in the skin. One notable pathway in skin aging is glycation, a process where sugars react with collagen and elastin to form advanced glycation end-products (AGEs) that stiffen fibers and cause yellowing and loss of elasticity. Ferulic acid has demonstrated anti-glycation effects: it can trap reactive carbonyl species (like methylglyoxal) and thereby prevent the formation of AGEs. Zheng et al. [66] reported that ferulic acid inhibits the formation of fluorescent AGEs and a major glycation product (CML—N(ε)-carboxymethyl lysine) in protein samples, while also reducing fructosamine levels. This ultimately helps prevent the collagen cross-linking and oxidative protein modifications that contribute to skin stiffening and sallowness in aged skin [66]. By blocking both glycation and oxidative stress, ferulic acid protects the skin’s support structures in a comprehensive way, targeting the main culprits of dermal aging.

Clinically, topical ferulic acid has been associated with improvements in wrinkles, fine lines, and overall skin youthfulness. In photoaged patients, use of ferulic acid either via peels or serums has yielded measurable anti-aging outcomes. Zduńska-Pęciak et al. [80] conducted a split-face trial comparing a ferulic acid peel versus an ascorbic acid peel on women with photoaged skin. Both sides showed significant reductions in fine lines and improvement in skin firmness, but the ferulic acid side was equally as effective as vitamin C in boosting elasticity and dermal density. In fact, adding ascorbic acid to the ferulic peel did not significantly enhance results, suggesting ferulic acid alone provided a robust stimulus for collagen remodeling [80]. Another study by the same group found that a series of 14% ferulic acid chemical peels led to notable improvements in skin hydration, elasticity, and reduction of wrinkle depth, with high patient satisfaction and minimal side effects [81]. These clinical improvements are consistent with ferulic acid’s ability to protect and stimulate the dermal matrix. By preserving collagen/elastin from degradation and possibly inducing some repair (due to reduced inflammation), the skin maintains a smoother and bouncier appearance.

Ferulic acid’s photoprotective actions (Section 5.1) also directly translate to anti-photoaging benefits. Regular use of ferulic acid under sunscreen or in day serums helps reduce the cumulative UV damage that leads to wrinkles, spots, and texture changes. For example, in a three-month study of women with signs of environmental aging (dullness, roughness), a ferulic acid antioxidant serum used daily resulted in significant improvements in skin texture, radiance, and fine line appearance compared to baseline [78]. The serum’s ability to neutralize urban pollution and UV-induced free radicals was credited for these anti-aging effects. Additionally, reductions in oxidative stress markers have been documented in skin treated with ferulic acid. One trial noted that a cream containing ferulic acid (along with vitamin C and phloretin) lowered UVA-induced lipid peroxidation in human skin ex vivo, correlating with less collagen damage in the treated samples [68]. Overall, ferulic acid helps skin to age more gracefully by protecting it from external aging factors and maintaining the integrity of its key structural proteins. It addresses both the extrinsic aging (photoaging, pollution) and some aspects of intrinsic aging (glycation, chronic inflammation), making it a valuable component of anti-aging skincare regimens.

### 5.5. Formulation and Delivery Considerations

Formulating ferulic acid into cosmetic products comes with some challenges and innovative solutions, especially to ensure stability and optimal delivery. FA in its pure form is susceptible to oxidation and can degrade with prolonged exposure to light, heat, or air. To harness its benefits in skincare, chemists have developed various strategies to stabilize ferulic acid and enhance its skin penetration.

#### 5.5.1. Optimal pH and Solvents

The solubility and ionization of FA are strongly influenced by its acidic nature (pKa ≈ 4.5) and by the predominance of the trans isomer, which is more stable than the cis form. Ferulic acid is most stable in acidic environments. Many ferulic-containing serums (such as those with vitamin C) use a pH around 3.0, which keeps FA in an unionized form that penetrates skin and resists degradation. Fortunately, FA’s skin absorption is not highly sensitive to pH changes: studies have shown that its penetration rate remains similar in formulations from pH 3 to 7. This gives formulators some flexibility. Typically, ferulic acid is dissolved in solvents like propylene glycol, ethanol, or glycerin in combination, which help solubilize the phenolic acid and facilitate its movement into the stratum corneum. The famous C + E + ferulic serum, for instance, uses 15% L-ascorbic acid (pH ~3) with 0.5% ferulic acid in an aqueous-alcohol base; the low pH stabilizes ascorbic acid, while ferulic acid remains stable and boosts the overall formula’s photostability [57].

#### 5.5.2. Encapsulation Technologies

Encapsulation is a key strategy to protect ferulic acid from premature degradation and to modulate its release into the skin. Recent research shows that loading ferulic acid into carriers like cyclodextrins, liposomes, or polysaccharide matrices can dramatically improve its stability. For example, Pueknang and Saewan [82] encapsulated ferulic acid in a phosphorylated rice starch polymer. In stress tests, the encapsulated FA retained ~96–98% of its content after exposure to high heat and UV light for 1 day, whereas free ferulic acid retained only ~67% under the same conditions. Over 15 days of light exposure, encapsulated FA showed about double the stability of free FA. This encapsulation not only preserved potency but also enhanced efficacy: a cream with the starch-encapsulated FA provided significantly greater skin-lightening and anti-wrinkle effects in a clinical trial than a cream with unencapsulated FA [82]. Encapsulation methods for ferulic acid include nanoemulsions, solid lipid nanoparticles (SLNs), nanostructured lipid carriers (NLCs), and chitosan or alginate microspheres. These systems shield FA from oxygen and UV until it is delivered into the skin. A 2023 study developed ferulic-acid-loaded nanostructured lipid carriers and showed that they improved both the delivery and stability of ferulic acid in a model skin system, compared to non-encapsulated ferulic acid [88]. Likewise, multiple emulsion systems (*w*/*o*/*w* emulsions) have been used to incorporate ferulic acid: Mancuso et al. [83] reported that a multiple emulsion protected ferulic acid from rapid oxidation and, when applied to human skin, led to better antioxidant activity in the tissue (and less UV erythema) than a conventional formulation [83].

#### 5.5.3. Derivative Forms

Another approach is to use ferulate derivatives that are more lipid-soluble or stable, which then release ferulic acid upon or after penetration. Examples include ethyl ferulate and ethylhexyl ferulate, ester forms of ferulic acid. These derivatives are often added to sunscreens and creams because they are oil-soluble and can act as UV filters themselves while gradually hydrolyzing to release active ferulic acid in the skin. Ethylhexyl ferulate in particular is used as a photostable antioxidant in sunscreens (it can synergize with other filters) [85]. Studies have shown that these ferulate esters retain the anti-inflammatory and antioxidant properties of ferulic acid, and they absorb UV radiation, but they are less prone to crystallization or discoloration in formulas [85]. One 2014 study demonstrated that a cream with ethyl ferulate had significant anti-inflammatory effects on skin and noted its advantage in formulation stability [67]. After topical application, skin esterases can convert a portion of these derivatives back into free ferulic acid, thus delivering the active molecule over time.

Ferulic acid has been successfully incorporated into a variety of cosmetic formats. Aside from the popular antioxidant serums (typically water- or glycol-based, for daily facial use) [57,73,74], ferulic acid is also used in creams and lotions for all-over photoprotection and anti-aging [72,78]. It is often found in day creams marketed for environmental defense [78]. Chemical peels with ferulic acid (at concentrations ~12–14%) have become a notable professional treatment for brightening and anti-aging—these peels are considered gentle yet effective, as ferulic’s antioxidant action reduces post-peel inflammation while the acid exfoliates [80,81]. Studies of ferulic acid peels show improvements in skin texture, pigmentation, and fine lines with minimal downtime [80,81]. Another novel format is dissolving microneedle patches containing ferulic acid or its combinations [84]. These tiny needle arrays can deliver ferulic acid into the deeper skin layers for targeted anti-wrinkle effects in areas like the eye wrinkles. Early trials reported enhanced skin quality (increased elasticity, reduced wrinkle depth) around the eyes after several weeks of applying ferulic acid microneedle patches, compared to placebo patches. Ferulic acid has also been formulated into spray mists, gels, and powders (encapsulated forms) for various cosmetic applications.

In formulating with ferulic acid, chemists must ensure photostability of the final product. Interestingly, ferulic acid can act as a stabilizer for other unstable ingredients (as noted with vitamin C). It has been observed that ferulic acid itself, when properly formulated (e.g., in a low-pH, airless pump, or dark bottle), remains effective over the typical shelf life of a cosmetic product. Many commercial products now include ferulic acid as a supporting antioxidant to boost overall formula resilience against light and heat. For instance, moisturizers or sunscreens might incorporate ferulic acid to scavenge any free radicals generated upon UV exposure of the formula, thereby protecting both the product and the skin.

## 6. Conclusions and Future Directions

Ferulic acid stands out as a versatile bioactive compound with implications for human health, food preservation, and cosmetics. Its strong antioxidant and anti-inflammatory properties support beneficial effects on components of metabolic syndrome, while also contributing to the stability and safety of foods and enhancing photoprotection and anti-aging in cosmeceutical formulations. Current evidence, particularly from preclinical models, is encouraging; however, larger and well-designed clinical trials are still needed to confirm efficacy in humans. At the same time, advances in delivery technologies to improve FA’s solubility and stability, as well as sustainable production approaches from agricultural by-products, represent key areas for future research. Continued interdisciplinary collaboration will be essential to unlock FA’s full potential and ensure its integration into innovative nutraceutical, functional food, and cosmetic products.

## Figures and Tables

**Table 1:** In vivo studies evaluating the effect of ferulic acid on the prevention and treatment of metabolic syndrome and associated disorders.

Disorder	Animal Model (*n* = Total Number of Animals)	Study Details	Experimental Findings	Reference
Obesity	Male C57BL/6J mice (*n* = 32)	HFD-induced obesity model; diet supplemented with FA (0.5% *w*/*w*) for 6 weeks.	FA significantly suppressed HFD-induced weight gain, visceral fat accumulation, adipocyte hypertrophy, and hepatic steatosis; it also improved serum lipid profile and glycemic control (comparable efficacy to the anti-obesity drug sibutramine).	[13]
Obesity	HFD-fed mice (*n* = 30)	Mice on high-fat diet with or without FA supplementation (0.5% of diet).	FA prevented HFD-induced increases in hepatic lipid accumulation and adipocyte size, accompanied by lower levels of pro-inflammatory cytokines (TNF-α, IL-6) in adipose tissue.	[14]
Dyslipidemia	ApoE^−/−^ mice (*n* = 32)	Atherosclerosis-prone mice on high-fat diet ± FA treatment (dose duration not specified).	FA lowered total cholesterol, triglycerides, and LDL-C; improved HDL/total cholesterol ratio; and significantly reduced aortic atherosclerotic plaque area (with enhanced collagen stability). Hepatic steatosis and liver enzymes were improved, comparable to the effects of simvastatin.	[15]
Dyslipidemia	Male mice (*n* = 40)	High-cholesterol diet ± FA (low and high dose) for 4 weeks.	FA supplementation attenuated diet-induced hypercholesterolemia (~13% lower serum cholesterol) by increasing hepatic bile acid synthesis via upregulation of CYP7A1, thereby promoting cholesterol catabolism and fecal excretion.	[16]
Dyslipidemia	ApoE^−/−^ mice (*n* = 24)	High-fat-diet-induced atherosclerosis with or without FA treatment.	FA upregulated brown adipose tissue UCP1 and induced “browning” of fat, increasing energy expenditure. Treated mice had significantly smaller aortic plaques and lower pro-inflammatory cytokine expression in lesions, indicating FA’s anti-atherosclerotic and anti-inflammatory effects.	[17]
Diabetes	Fructose/STZ-induced type 2 diabetic rats (*n* = 32)	Rats rendered diabetic with fructose diet + STZ; treated with FA (150 or 300 mg/kg/day) for 5 weeks.	FA significantly reduced hyperglycemia and improved serum insulin levels in diabetic rats, reflecting enhanced insulin action and glycemic control.	[18]
Diabetes	HFD-induced diabetic mice (*n* = 24)	Obese insulin-resistant mice on HFD with or without FA treatment.	FA improved glucose tolerance and insulin sensitivity, associated with suppression of hepatic gluconeogenic enzymes (PEPCK, G6Pase), and increased hepatic glycogen storage. FA’s activation of insulin signaling (e.g., PI3K/Akt–FoxO1) in liver led to reduced hepatic glucose output and better glycemic control.	[19]
Diabetes	STZ-induced diabetic rats (*n* = 24)	Type 1 diabetic rats treated with FA (dose and duration as per study).	FA increased GLUT4 expression and translocation in insulin-sensitive tissues. In diabetic hearts, FA treatment upregulated myocardial GLUT4 and PI3K/Akt activity, improving cardiac glucose uptake and overall insulin responsiveness.	[20]
Hypertension	Spontaneously hypertensive rats (*n* = 45)	Experiment 1: single oral dose of FA (30–600 mg/kg); experiment 2: diet containing 0.5% FA for 8 weeks.	A single administration of FA acutely lowered blood pressure in SHR (minimum effective dose ~100 mg/kg). Long-term dietary FA intake attenuated the development of hypertension, as FA-fed young SHR showed significantly reduced blood pressure gains over 6–8 weeks compared to controls.	[21]
Metabolic syndrome	Male C57BL/6J mice (*n* = 50)	HFD-induced metabolic syndrome model with interventions: exercise (Ex), FA (100 mg/kg BW oral), or combined (Ex + FA), 13 weeks.	FA plus regular exercise produced greater benefits than either alone: the combo prevented HFD-induced weight gain and adiposity, improved serum lipid profile, enhanced hepatic antioxidant enzyme activities, and improved exercise endurance more than FA or exercise by itself.	[22]

Abbreviations: ApoE^−/−^ = apolipoprotein E knockout; HFD = high-fat diet; STZ = streptozotocin; BW = body weight; LDL-C = low-density lipoprotein cholesterol; HDL-C = high-density lipoprotein cholesterol; UCP1 = uncoupling protein 1; PI3K = phosphatidylinositol-3-kinase; Akt = protein kinase B; PEPCK = phosphoenolpyruvate carboxykinase; G6Pase = glucose-6-phosphatase.

**Table 2:** Clinical trials evaluating the effect of ferulic acid or ferulic-acid-rich foods and supplements on the prevention and treatment of metabolic syndrome and associated disorders.

Disorder	FA Source	Subjects (*n* = Total)	Study Details	Experimental Findings	Reference
Obesity	Brown rice (whole grain, FA-rich)	Overweight adults (*n* ≈ 50)	12-week dietary intervention replacing white rice with brown rice (high ferulic acid content).	Modest but significant decreases in body weight, BMI, and waist circumference were observed in the brown rice group compared to the white rice group.	[23]
Obesity	Brewer’s spent grain extract (rich in FA)	Prediabetic adults (*n* ≈ 40)	Randomized cross-over trial of a ferulic-acid-rich brewer’s spent grain supplement vs. placebo.	Body weight remained stable (no gain) in both groups, but the FA-rich supplement led to improved inflammatory profiles (significantly lower levels of inflammatory markers) in participants.	[23]
Dyslipidemia	Pure ferulic acid (supplement)	Hyperlipidemic subjects (*n* = 48)	6-week double-blind RCT: FA 1000 mg per day vs. placebo.	FA supplementation significantly reduced total cholesterol (~8%↓), LDL-C (~9%↓) and triglycerides (~12%↓), with a slight increase in HDL-C (~4%↑). FA also lowered oxidized LDL and markers of oxidative stress and inflammation (e.g., ~33%↓ in CRP), improving overall cardiovascular risk profile.	[24]
Diabetes	γ-Oryzanol–fortified canola oil (ferulate ester-rich)	Adults with type 2 diabetes (*n* = 92)	12-week RCT comparing daily use of γ-oryzanol enriched canola oil vs. regular canola or sunflower oil.	Only the ferulic-rich oil group showed significant improvements: fasting triglycerides dropped by ~17.9 mg/dL, and notable reductions in waist circumference, blood pressure, fasting glucose, and HbA_1c_ were achieved. (No significant lipid changes in control oil groups.) These results highlight the efficacy of FA derivatives in improving multiple MetS components.	[25]
Hypertension	Wheat-aleurone-rich diet (high in FA)	Overweight individuals at MetS risk (*n* = 23)	8-week cross-over diet trial: whole-grain wheat aleurone diet vs. refined wheat diet.	No significant changes in blood pressure were observed over 8 weeks. However, the FA-rich aleurone diet markedly improved oxidative stress indices—urinary 8-iso-prostaglandin F2α (8-isoprostane) excretion decreased by ~33%—indicating enhanced antioxidant status despite no acute BP reduction.	[26]

Abbreviations: BMI = body mass index; LDL-C = low-density lipoprotein cholesterol; HDL-C = high-density lipoprotein cholesterol; CRP = C-reactive protein; RCT = randomized controlled trial; HbA_1c_ = glycated hemoglobin (measure of long-term glycemic control); MetS = metabolic syndrome.

**Table 3:** Key studies on ferulic acid (FA) as a food additive, highlighting antimicrobial, antioxidant, and prebiotic effects.

Study Type	Food Matrix or Model System	FA Form or Delivery Method	Main Observed Effect	Reference
In vitro (broth culture) and food application (meat model)	Ready-to-eat cold-cut meat inoculated with *E. coli* O157:H7 and *L. monocytogenes*	FA combined with caffeic acid (each 150–200 ppm)	Synergistic antibacterial action: ~3.6 log CFU/g reduction of *E. coli* O157:H7 on meat at 4 °C in 72 h with FA + caffeic (greater than either alone); also significantly inhibited *L. monocytogenes*, improving pathogen control in cold cuts.	[38]
In vitro (culture MIC assay) and food application (seafood preservation)	*Shewanella putrefaciens* culture; refrigerated crayfish (*Procambarus clarkii*) tails	FA + ε-polylysine (each at ¼ MIC); applied in storage (with plasma-activated water)	Synergistic inhibition of spoilage bacteria: FA + ε-polylysine completely stopped *S. putrefaciens* growth in vitro at quarter doses (each alone ineffective). In refrigerated crayfish, the combination slowed spoilage—lower total volatile bases and curtailed fishy off-odor compounds—thereby extending shelf life.	[39]
Food application (challenge test in beverage)	Fresh apple and orange juices (inoculated with *L. monocytogenes*)	FA fortification (1500 mg/L added to juices)	Inhibited *L. monocytogenes* growth during 4 °C storage: FA-fortified juices had significantly lower Listeria counts after 9 days vs. control, while native juice microbiota were largely unaffected. FA increased juice antioxidant content but caused slight acidification and color change (minor sensory impact).	[40]
Food application (dairy product)	High-moisture fresh cheese (e.g., Queso fresco) inoculated with *L. monocytogenes*	FA incorporated into cheese (alone or with nisin)	Suppressed *L. monocytogenes* in cheese during refrigeration; FA (± nisin) inhibited Listeria growth and prevented development of resistant subpopulations. Listeria exposed to sublethal FA did not acquire increased tolerance, suggesting low risk of resistance. FA (with nisin) is an effective hurdle to improve cheese safety.	[41]
Food application (edible coating)	Refrigerated sea bass fillets (fish)	Edible coating of konjac glucomannan + ε-polylysine + FA	Improved microbial quality and shelf life: fish fillets coated with the FA/ε-PL composite film had significantly lower total viable bacterial counts during 4 °C storage vs. controls. FA in coating inhibited spoilage bacteria like *S. putrefaciens*, delaying spoilage (lower TVB-N accumulation) and preserving fresh odor longer.	[42]
In vitro (food model system)	Oil-in-water emulsion (simulated food matrix) inoculated with *L. monocytogenes*	Free FA (as antimicrobial additive) compared to eugenol	Maintained anti-Listeria activity in an emulsion: FA continued to effectively inhibit *L. monocytogenes* in an O/W emulsion, whereas the more lipophilic eugenol lost efficacy due to partitioning into the oil phase. Demonstrates FA’s advantage in emulsified/high-fat foods for pathogen control.	[43]
Food application (meat preservation)	Dried beef product (shelf-stable meat snack)	FA added at 0.1% (*w*/*w*) in formulation	Strong antioxidant protection: 0.1% FA markedly suppressed lipid oxidation during storage—TBARS remained below sensory rancidity threshold over 6 months (no rancid off-flavors), whereas control showed oxidation by 2 months. FA-treated meat had significantly lower peroxide values and sustained higher antioxidant capacity, effectively extending oxidative shelf life.	[44]
In vitro (oil oxidation assay)	Bulk cold-pressed flaxseed oil (rich in ω-3 linolenic acid)	FA added to oil (0.02–0.1%)	Enhanced oxidative stability of oil: FA prolonged the induction time for oxidation in a dose-dependent manner. Even low levels (≤0.1%) delayed peroxide formation and rancidity, extending oil shelf life compared to untreated oil.	[45]
Food application (encapsulated oil)	Fish oil encapsulated in electrospun zein fiber mat	FA incorporated into encapsulating fiber matrix	Protection of highly unsaturated lipids: FA in the zein nanofiber walls significantly slowed oxidation of the encapsulated fish oil during storage. Omega-3-rich oil with FA showed reduced peroxidation without negative effects on oil release or sensory properties, indicating improved stability of functional food oils.	[46]
In vitro (model membrane and cell assays)	Liposomal membranes and cultured cells (oxidative stress model)	Free FA (alone and combined with vitamins C and E)	Antioxidant efficacy and synergy: FA was the most effective single antioxidant in protecting model membranes from lipid peroxidation, and it synergistically enhanced the protection by α-tocopherol (vitamin E) and ascorbic acid (vitamin C). FA stabilized the radicals of vitamins C/E, regenerating their active forms, thus doubling the overall antioxidant effect compared to vitamins alone.	[47]
Food application (beverage color stability)	Model beverages colored with elderberry (Sambucus) and purple carrot anthocyanins	FA added as copigment (in solution)	Improved color intensity and stability: FA addition yielded a hyperchromic effect (deeper initial color) and slowed anthocyanin degradation during 8-week storage. Drinks with FA maintained higher color saturation and anthocyanin levels over time versus controls, demonstrating pigment stabilization by copigmentation.	[11]
In vitro (anthocyanin stability test)	Anthocyanin solutions from blackcurrant pomace	FA added as copigment	Extended thermal and pH stability of pigments: copigmentation with FA increased the half-life of blackcurrant anthocyanins at moderate pH (~6) and under heat stress. Ferulic-treated anthocyanins showed significantly better color retention vs. no copigment, indicating enhanced pigment stability at elevated temperature.	[48]
In vitro (anthocyanin stability test)	*Hibiscus sabdariffa* (roselle) anthocyanin extract	FA added as copigment	Higher heat stability of natural colorant: FA incorporation significantly improved the thermal stability of Hibiscus anthocyanins, resulting in less color loss under high-temperature conditions. This suggests FA can help preserve color in heat-processed foods and beverages by protecting anthocyanin pigments.	[49]
Food application (edible film on produce)	Fresh-cut apple slices	Soy-protein-isolate-based edible coating with added FA	Improved coating performance and product quality: inclusion of FA in the soy protein coating led to cross-linking that enhanced the film’s tensile strength and water resistance. The stronger, antioxidant-infused coating likely slowed apple browning and moisture loss, thereby preserving the fresh quality of cut apples.	[50]
In vivo (animal study)	High-fat diet (HFD)-induced obese mice (12-week study)	Oral FA supplementation (100 mg/kg body weight per day)	Modulation of gut microbiota and metabolites: FA shifted the gut microbiome toward beneficial groups—increased SCFA-producing bacteria (e.g., *Faecalibaculum*, *Olsenella*) and reduced endotoxin-producing taxa. Consequently, colonic SCFA levels (especially butyrate) rose and gut barrier function improved (higher tight junction protein, lower LPS), leading to reduced inflammation. Overall metabolic health markers (insulin sensitivity, hepatic fat) were ameliorated via this microbiota modulation.	[28]
In vivo (animal study)	ApoE^−^/^−^ mice (atherosclerosis-prone model)	FA supplementation (diet or gavage, dose as per study)	Altered gut microbiome linked to metabolic benefit: FA treatment reshaped gut microbial composition and fecal metabolites in hyperlipidemic mice, which was associated with downregulation of hepatic SREBP-1 and reduced lipogenesis. The microbiota changes (e.g., enrichment of butyrate producers) contributed to attenuated atherosclerotic injury and improved lipid metabolism in this model.	[15]
In vivo (human dietary intervention)	Adult volunteers on a wheat bran/aleurone-rich diet vs. refined wheat diet (8 weeks)	Diet naturally high in bound FA (in cereal fiber)	Prebiotic fermentation and SCFA boost: consuming the FA-rich wheat aleurone diet increased total fecal short-chain fatty acid output and shifted SCFA profile toward higher butyrate proportion, compared to a low-FA refined diet. This indicates enhanced colonic fermentation of ferulate fiber, supporting gut health (butyrogenesis) in humans.	[51]
In vitro (fecal culture) and human microbiome analysis	Wheat bran fermentation by human gut microbiota (laboratory batch culture); also observed in vivo in humans consuming bran	Bound FA present in wheat bran fiber (released by gut microbes)	Enrichment of butyrate-producing bacteria: fermentation of wheat bran (rich in ferulate) promoted growth of butyrogenic gut bacteria that liberate FA from fiber. This microbial shift leads to increased butyrate generation in the colon, contributing to gut health and explaining the prebiotic effect of ferulic-containing fiber.	[52]

**Table 4:** Preclinical and clinical studies on ferulic acid in cosmetic applications. The table compiles key in vitro (cell-based), in vivo (animal), and clinical studies, including model/subjects, formulation, and main skin effects (photoprotection, anti-aging, anti-inflammatory, skin brightening, wound healing).

Study Type	Model/Subjects	Application or Formulation	Main Effects	Reference
In vitro (enzyme and cell)	Tyrosinase enzyme assay; B16 melanoma cells (mouse)	Ferulic acid (free) added in solution (comparative to caffeic acid)	Competitive inhibition of tyrosinase activity, reducing melanin synthesis. In B16 cells, ferulic acid significantly lowered melanin content without cytotoxicity, indicating a depigmenting effect.	[64]
In vitro (cell culture)	Human dermal fibroblasts	Ferulic acid pre-treatment before UVA irradiation	Protected skin cells from UVA-induced damage: fibroblasts showed higher viability and reduced collagenase (MMP) activity after UVA exposure with ferulic acid, compared to untreated cells.	[65]
In vitro (biochemical)	Protein glycation assay (BSA + sugar)	Ferulic acid added to protein/sugar reaction mixture	Inhibited formation of advanced glycation end-products (AGEs, e.g., CML) and lowered fructosamine levels. This anti-glycation activity suggests ferulic acid helps prevent collagen cross-linking (anti-aging).	[66]
In vitro (formulation efficacy)	Emulsion formulation testing (lab-based)	Cream containing ethyl ferulate (ferulic acid derivative)	Demonstrated significant anti-inflammatory effects in a skin model (reduced inflammation markers) and improved formulation stability versus free ferulic acid. After topical application, skin esterases slowly convert the ester to active ferulic acid.	[67]
Ex vivo (human skin)	Human skin explants (laboratory setting)	Cream with ferulic acid + vitamin C + phloretin (antioxidants)	Lowered UVA-induced oxidative damage: treated skin had significantly less lipid peroxidation and collagen damage after UVA exposure compared to untreated controls.	[68]
Ex vivo (skin model)	Artificial/excised skin model	Ferulic acid in nanostructured lipid carriers (NLC) vs. free FA	Enhanced delivery and stability of ferulic acid: the NLC formulation increased skin penetration and protected FA from degradation, compared to non-encapsulated ferulic acid.	[43]
In vivo (animal)	Diabetic rats (wound-healing model)	Topical ferulic acid treatment on excisional wounds	Accelerated wound closure and repair: ferulic-acid-treated wounds healed faster with enhanced granulation tissue formation and collagen deposition, while excess inflammation was reduced.	[69]
In vivo (animal)	Mice (UVB-induced photoaging model)	Topical ferulic acid during chronic UVB exposure	Photoprotection against photoaging: ferulic acid application suppressed UV-induced MMP-2 and MMP-9 upregulation, preventing collagen degradation; treated mice had less collagen damage and wrinkle formation than UV-exposed controls.	[70]
In vivo (animal)	Mice (atopic dermatitis model)	Topical ferulic acid on irritated skin	Anti-inflammatory and barrier-protective effects: alleviated dermatitis symptoms (reduced redness, swelling, epidermal thickening) by downregulating pro-inflammatory cytokines (IL-6, TNF-α). Treated mice showed improved skin barrier integrity and less immune cell infiltration.	[71]
Clinical (human volunteer study)	Healthy human volunteers (in vivo SPF testing)	Broad-spectrum sunscreen + 1% ferulic acid vs. sunscreen alone	Enhanced photoprotection: adding ferulic acid increased sun protection factor (SPF) by ~37% and UVA-PF by ~26% compared to the same sunscreen without FA. Ferulic acid also reduced UV-induced skin erythema (redness), indicating added anti-inflammatory benefits.	[72]
Clinical (controlled trial)	Human volunteers (controlled UV exposure study)	Topical antioxidant serum (15% vitamin C + 1% vitamin E + 0.5% FA) pre-UV	Synergistic photoprotection with vitamins C + E: the ferulic acid serum doubled the photoprotection of skin compared to vitamins C + E alone. Skin pre-treated with the C + E + FA serum showed significantly less UVB-induced erythema and fewer sunburn cells versus untreated skin.	[57]
Clinical (controlled trial)	Human volunteers (controlled UV exposure study)	Topical vitamin C + E + ferulic acid serum (same as above)	Confirmed enhanced photoprotection (replicating: pre-treatment with the C + E + FA serum led to significantly reduced UVB-induced erythema and sunburn cell formation compared to no antioxidant treatment, demonstrating ferulic acid’s crucial role in the serum’s efficacy.	[73]
Clinical (double-blind RCT)	Human subjects (face; photodamage protection)	Facial serum (15% vitamin C + 1% vitamin E + 0.5% FA) vs. placebo	Protection against acute UV photodamage: after controlled UV exposure, subjects using the C + E + FA serum had significantly less skin redness by day 4 compared to controls, indicating superior photoprotection in a real-world scenario.	[74]
Clinical (controlled trial)	Patients after ablative laser resurfacing	Post-procedure use of ferulic acid antioxidant serum vs. no serum	Accelerated wound healing and reduced hyperpigmentation: the ferulic acid serum (with vitamins C/E) sped up post-laser skin recovery—patients had improved texture and significantly less post-inflammatory hyperpigmentation (PIH) than controls—without increasing irritation.	[75]
Ex vivo + Clinical (patch test)	Ex vivo human skin; healthy volunteers (patch test)	Sunscreen + ferulic acid vs. sunscreen without FA	Anti-inflammatory benefits in formulations: in ex vivo skin, the FA-enriched sunscreen showed lower UV-induced inflammatory markers than the FA-free version. In human patch tests, skin treated with FA-sunscreen had significantly less chemically induced redness (from an irritant) compared to control, demonstrating ferulic acid’s soothing, anti-inflammatory effect.	[76]
Clinical (RCT)	Patients with mild-to-moderate rosacea	Topical ferulic acid added to standard therapy vs. standard alone	Anti-inflammatory and barrier repair in rosacea: after 6 weeks, the ferulic acid group showed greater reduction in inflammatory lesions and redness, plus improved skin barrier function (lower transepidermal water loss, higher hydration) compared to controls. Ferulic acid was well tolerated (minimal irritation) and hastened symptom relief.	[77]
Clinical (single-blind trial)	Women in urban high-pollution area (3-month study)	Daily antioxidant serum (Deschampsia antarctica extract + ferulic acid + vitamin C)	Skin-brightening and anti-aging effects: at 4 weeks, the ferulic acid serum significantly reduced dark spots and improved skin brightness; by 3 months, it enhanced skin texture, radiance, and reduced fine lines. These benefits are attributed to the serum’s photoprotective and anti-pollution antioxidant action.	[78]
Clinical (split-face study)	Patients with melasma (facial hyperpigmentation)	Ferulic-acid-containing formula on one side of face vs. alternate treatment on other side	Skin lightening in melasma: the side treated with ferulic acid showed a greater decrease in melanin index and pigment intensity than the side treated with the comparative regimen, leading to more pronounced fading of melasma patches (both treatments produced improvement).	[79]
Clinical (split-face trial)	Women with photoaged skin	14% ferulic acid chemical peel on one side vs. 14% ascorbic acid (vitamin C) peel on the other	Anti-aging (collagen remodeling): both peels improved fine wrinkles and firmness, but the ferulic acid peel was as effective as the vitamin C peel in increasing skin elasticity and dermal density. Notably, adding vitamin C to a ferulic acid peel yielded no further benefit, indicating ferulic acid alone robustly stimulated collagen renewal.	[80]
Clinical (open-label series)	Individuals with signs of skin aging	Series of 14% ferulic acid chemical peels (multiple sessions)	Anti-aging outcomes: the ferulic acid peel series produced marked improvements in skin hydration and elasticity and a reduction in wrinkle depth. Patients reported high satisfaction with visible results and experienced minimal side effects, underscoring the peels’ safety and efficacy.	[81]
Clinical (product comparison)	Human volunteers (skincare efficacy trial)	Topical cream with encapsulated ferulic acid (in phosphorylated starch) vs. cream with free ferulic acid	Improved efficacy via encapsulation: the starch-encapsulated ferulic acid cream provided significantly greater skin-lightening and anti-wrinkle effects than an identical cream with unencapsulated FA. Encapsulation also conferred higher stability of FA under stress conditions, enhancing overall formulation performance.	[82]
Clinical (formulation test)	Healthy volunteer skin (UV exposure test sites)	Ferulic acid in multiple emulsion (*w*/*o*/*w*) vs. conventional formulation	Enhanced photoprotection with advanced formulation: the multiple-emulsion formulation protected ferulic acid from oxidation and, when applied to skin, led to higher tissue antioxidant levels and significantly less UV-induced erythema compared to a standard (non-encapsulated) formulation.	[83]
Clinical (pilot trial)	Adults with periocular (eye-area) wrinkles	Dissolving microneedle patches with ferulic acid (vs. placebo patches)	Targeted anti-wrinkle efficacy: after several weeks of use, the ferulic acid microneedle patches significantly increased skin elasticity and reduced wrinkle depth in the crow’s-feet area compared to placebo, demonstrating the novel delivery method’s effectiveness.	[84]

## Data Availability

No new data were created or analyzed in this study.
